# Broadening horizons: the multifaceted functions of ferroptosis in kidney diseases

**DOI:** 10.7150/ijbs.85674

**Published:** 2023-07-16

**Authors:** Qi Feng, Yang Yang, Kaidi Ren, Yingjin Qiao, Zhi Sun, Shaokang Pan, Fengxun Liu, Yong Liu, Jinling Huo, Dongwei Liu, Zhangsuo Liu

**Affiliations:** 1 Research Institute of Nephrology, Zhengzhou University, the First Affiliated Hospital of Zhengzhou University, Zhengzhou 450052, P. R. China; 2 Traditional Chinese Medicine Integrated Department of Nephrology, the First Affiliated Hospital of Zhengzhou University, Zhengzhou 450052, P. R. China; 3 Henan Province Research Center for Kidney Disease, Zhengzhou 450052, P. R. China; 4 Key Laboratory of Precision Diagnosis and Treatment for Chronic Kidney Disease in Henan Province, Zhengzhou 450052, P. R. China; 5 Clinical Systems Biology Laboratories, the First Affiliated Hospital of Zhengzhou University, Zhengzhou, 450052, P. R. China; 6 Department of Pharmacy, the First Affiliated Hospital of Zhengzhou University, Zhengzhou 450052, P. R. China.; 7 Blood Purification Center, the First Affiliated Hospital of Zhengzhou University, Zhengzhou 450052, P. R. China.

**Keywords:** ferroptosis, kidney diseases, molecular mechanism, pharmacological progress

## Abstract

Ferroptosis is an iron-dependent programmed cell death pattern that is characterized by iron overload, reactive oxygen species (ROS) accumulation and lipid peroxidation. Growing viewpoints support that the imbalance of iron homeostasis and the disturbance of lipid metabolism contribute to tissue or organ injury in various kidney diseases by triggering ferroptosis. At present, the key regulators and complicated network mechanisms associated with ferroptosis have been deeply studied; however, its role in the initiation and progression of kidney diseases has not been fully revealed. Herein, we aim to discuss the features, key regulators and complicated network mechanisms associated with ferroptosis, explore the emerging roles of organelles in ferroptosis, gather its pharmacological progress, and systematically summarize the most recent discoveries about the crosstalk between ferroptosis and kidney diseases, including renal cell carcinoma (RCC), acute kidney injury (AKI), diabetic kidney disease (DKD), autosomal dominant polycystic kidney disease (ADPKD), renal fibrosis, lupus nephritis (LN) and IgA nephropathy. We further conclude the potential therapeutic strategies by targeting ferroptosis for the prevention and treatment of kidney diseases and hope that this work will provide insight for the further study of ferroptosis in the pathogenesis of kidney-related diseases.

## Introduction

Kidney diseases have become a prominent threat of human death. At present, there are approximately 850 million patients with kidney diseases worldwide, and the number of people suffering from kidney disease in China has exceeded 100 million^1^. Epidemiological statistics recently reported that the global prevalence rate of chronic kidney disease (CKD) is over 10%^2^. The pathogenesis of kidney diseases is very complex and involves the interaction of multiple key factors and mechanisms, among which kidney injury caused by cell death plays an essential role in driving the development of various kidney diseases^3^.

Regulated cell death is indispensable for maintaining normal physiological homeostasis, and either excessive or insufficient cell death can lead to the initiation of diseases^4^. Emerging evidence indicates that multiple forms of cell death, including apoptosis, autophagy, necroptosis and pyroptosis^5^, are involved in the pathogenesis of kidney diseases^6^. Since ferroptosis, a new form of cell death driven by iron-dependent lipid peroxidation, was first proposed by Stockwell et al. in 2012^7^, it has been proven to participate in the occurrence and development of a variety of human diseases, including kidney diseases^8^.

The pathophysiological relevance of ferroptosis was first found in acute kidney injury (AKI) and renal cell carcinoma (RCC). Since then, a growing body of evidence has reported that ferroptosis plays a core role in multiple kidney diseases, such as CKD and lupus nephritis (LN)^9^-^12^. In recent years, plenty of reports have suggested that targeting the ferroptosis of renal cells may provide a highly available strategy for treating patients with kidney diseases.

In this review, we browse and summarize the current research progress considering the role of ferroptosis in different types of kidney diseases. We discuss the characteristics, potential mechanisms and pharmacological progress of ferroptosis, further elucidate the disorders of iron and lipid metabolism encountered in kidney diseases and describe their potential connection with ferroptosis. We hope this review will provide new perspectives regarding the pathological role of ferroptosis in kidney diseases.

## Overview of ferroptosis

Early classifications of cell death mainly refer to apoptosis, autophagy, necrosis, and pyroptosis. However, over time, a variety of new cell death models have been proposed, such as ferroptosis^7^, ferritinophagy^13^ and cuproptosis^14^, among which ferroptosis is being widely explored. Compared to other types of cell death, ferroptosis is characterized by membrane lipid peroxidation caused by intracellular iron overload and increased reactive oxygen species (ROS) (**Table 1**). Morphologically, it is mainly characterized by mitochondrial atrophy, increased membrane density and decreased or absent mitochondrial cristae. Biochemically, it is mainly glutathione depletion and GPX4 activity decline. Genetically, ferroptosis-related mechanisms are complex and mediated by multiple genes, mainly involving gene changes in iron homeostasis, lipid peroxidation and amino acid metabolism. Although the dysregulation of iron metabolism and lipid metabolism is closely related to ferroptosis, it still has no specific molecular indicators at present, except for detecting the levels of intracellular lipid peroxides and the occurrence of cell death inhibited by ferroptosis inhibitors or iron chelators^15^.

## The main regulatory mechanisms of ferroptosis

**Figure 1:**
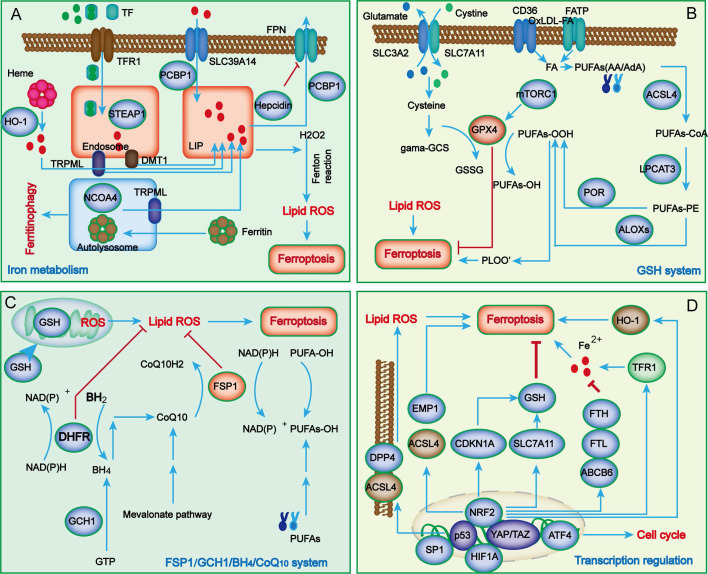
** The main regulatory mechanisms of ferroptosis**. Iron metabolism abnormalities are the foundation of ferroptosis (A). When iron overload occurs, excess iron is stored in ferritin, and free iron participates in inducing ROS generation through the Fenton reaction. Cellular antioxidant systems eliminating lipid peroxidation of membrane phospholipids mainly function through the system Xc^-^/GPX4 axis (B) and other parallel metabolic pathways, including the FSP1/CoQ_10_/NADPH system, mitochondria DHODH/CoQH_2_ system, and GCH1/BH4/DHFR system (C). Recently, several key transcription regulators, such as p53, Nrf2, ATF4, SP1, HIF-1A and YAP/TAZ, have been confirmed to regulate ferroptosis (D). ABCB6, ATP-binding cassette subfamily B member 6; LOXs, lipoxygenases; ATF4, activating transcription factor 4; BACH1, BTB domain and CNC homolog 1; BH_2_, 7,8-dihydrobiopterin; BH_4_, tetrahydrobiopterin; CDKN1A, cyclin-dependent kinase inhibitor p21; CHMP5/6, chromatin modeling protein 5/6; CoQ_10_H_2_, ubiquinol; GCH1, guanosine triphosphate cyclohydrolase 1; DHFR, dihydrofolate reductase; DHODH, dihydroorotate dehydrogenase; DDP4, dipeptidyl peptidase-4; DMT1, divalent metal transporter1; EMP1, epithelial membrane protein 1; ESCRT-III, endosomal sorting complex required for transport III; FPN, Ferroportin; FSP1, ferroptosis suppressor protein 1; FTH, ferritin heavy chain; FTL, ferritin light chain; GCS, glutamylcysteine synthetase; GCH1, guanosine triphosphate cyclohydrolase 1; GSH, glutathione; HO-1, heme oxygenase 1; LIP, labile iron pool; LPCAT3, lysophosphatidylcholine acyltransferase 3; MTX, methotrexate; mTORC1, mechanistic target of rapamycin complex 1; PCBP, poly (RC)-binding proteins; POR, NADPH-cytochrome P450 reductase;; STEAP3, six-transmembrane epithelial antigen of prostate 3; TXNRD1, thioredoxin reductase 1; TRPML1/2, Mucolipin TRP channel 1/2.

In contrast to other types of cell death, the characteristics of ferroptosis mainly manifest as iron overload and lipid peroxide accumulation. During the process of renal injury, in addition to the classical abnormal regulation of iron metabolism and lipid peroxidation, a growing number of novel metabolic pathways have also been uncovered in ferroptosis, such as the system Xc^-^/glutathione peroxidase 4 (GPX4) system, ferroptosis suppressor protein 1 (FSP1)/ubiquinol (CoQ10) system, dihydroorotate dehydrogenase (DHODH)/dihydroubiquione (CoQH_2_) system, and GTP cyclohydrolase 1 (GCH1)/tetrahydrobiopterin (BH4)/dihydrofolate reductase (DHFR) system (**Figure 1**).

### Iron metabolism

Iron is an important mediator in many physiological activities in mammals. Iron deficiency leads to iron deficiency anemia, whereas its overload can produce abundant ROS and further cause oxidative damage in tissues or organs, such as kidney injury^16^. Therefore, cellular and systemic iron homeostasis is tightly regulated to maintain the redox balance.

Under common conditions, systemic Fe^3+^ can be transformed into Fe^2+^ through transferrin (TF) and transferrin receptor 1 (TFR1) and then released into the labile iron pool (LIP) of the cytoplasm, while excessive iron is stored in ferritin (FT). As the key component of FT, ferritin heavy chain 1 (FTH1) has ferroxidase activity and can catalyze the conversion of Fe^2+^ to Fe^3+17^. In the case of abnormal iron transport, the upregulation of TFR1 and downregulation of FTH1 lead to the overload of Fe^2+^. Subsequently, excessive Fe^2+^ reacts with hydrogen peroxide to produce hydroxyl radicals, which can directly catalyze the formation of lipid peroxides through the Fenton reaction and eventually promote cell ferroptosis^10^. Although iron overload is a major characteristic of ferroptosis, its potential regulatory mechanisms are still unknown. Many studies have revealed that iron overload might induce abnormal mitochondrial oxidative phosphorylation and abundant ROS production, thereby oxidizing polyunsaturated fatty acids (PUFAs) on cell and organelle membranes to form lipid peroxides, which further directly or indirectly destroy cell integrity and eventually trigger ferroptosis.

### Lipid peroxidation

Lipid peroxidation refers to the process by which PUFAs are oxidized by ROS via enzymatic or nonenzymatic reactions to form lipid peroxides, which is closely related to ferroptosis^18^. Arachidonic acid (AA) is one of the most frequently consumed PUFAs during ferroptosis and can be esterified into phosphatidyl ethanolamine (PE) under the catalysis of acyl CoA synthase long-chain family member 4 (ACSL4) and lysophosphatidylcholine acyltransferase 3 (LPCAT3) and further oxidized into toxic lipid peroxides by arachidonic acid lipoxygenases (ALOXs)^19^-^21^. ACSL4 and LPCAT3 are closely related to the biosynthesis and remodeling of PE. ALOXs are nonheme iron-containing enzymes that catalyze the oxidation of PUFAs to produce malondialdehyde (MDA) and 4-hydroxynonenal (4-HNE), thereby promoting ferroptosis^22^. Taken together, since the excess activation of ACSL4, LPCAT3 and ALOXs are considered key drivers of ferroptosis, decreasing their expression will reduce the accumulation of lipid peroxides, thus inhibiting ferroptosis progression.

### System Xc-/glutathione peroxidase 4 (GPX4) axis

Glutathione (GSH) is the main intracellular antioxidant in mammals and is synthesized by glutamate, cysteine and glycine under the catalysis of glutamine cysteine ligase (GCL) and glutathione synthesis (GSS)^23^. Because the intracellular concentration of cysteine is limited, it is regarded as the rate-limiting precursor during GSH synthesis. Notably, cysteine cannot enter cells through passive transport and only relies on the specific transporter system Xc^-^.

System Xc^-^ is composed of transporter protein solute carrier family 7 member 11 (SLC7A11) and solute carrier family 3 member 2 (SLC3A2), which can transport intracellular glutamate to the outside and uptake extracellular cystine into cells to reduce it to cysteine^7^. Studies have shown that erastin and sulfasalazine can interfere with cystine absorption by inhibiting system Xc- activity, causing a reduction in GSH synthesis and a decrease in GPX4 activity, thereby inducing lipid peroxidation and cell death 24. Mice lacking SLC7A11 undergo cell death, while this condition can be ameliorated by administration of ferroptosis inhibitors^25^, ^26^; however, upregulation or overexpression of SLC7A11 can also achieve similar results. Therefore, SLC7A11 is widely accepted as an executor of ferroptosis.

GPX4 is a GSH-dependent enzyme that can convert reduced glutathione to oxidized glutathione (GSSG) and reduce lipid hydroperoxide (L-OOH) to lipid alcohol (L-OH) to protect against lipid peroxidation. As a well-characterized ferroptosis suppressor, GPX4 can inhibit the accumulation of iron-dependent lipid peroxides and participates in modulating a variety of physiological functions^27^. Studies have found that *Gpx4* knockout in mice causes embryonic lethality, whereas conditional knockout of *Gpx4* leads to dysfunctions of the immune system, liver, kidney, brain and other tissues and organs in mice^28^. Therefore, blocking GSH synthesis or inactivating system Xc^-^ will reduce GPX4 activity and limit cell antioxidant capacity, ultimately resulting in ferroptosis. Therefore, maintaining metabolic homeostasis of the system Xc^-^/GPX4 axis might be a core factor in counteracting ferroptosis.

### Ferroptosis suppressor protein 1 (FSP1)/ubiquinol (CoQ10)/NADPH system

In addition to the system Xc^-^/GPX4 axis, genome-wide screening has recently identified FSP1 as another ferroptosis suppressor^29^. As an alternative ferroptosis suppressive mechanism, the FSP1/CoQ_10_/NADPH system is independent of the intracellular expression levels of GSH, GPX4 and ACSL4 and can protect cells from GPX4 inhibition-induced ferroptosis^30^. FSP1 is mainly distributed in lipid droplets and plasma membranes and can inhibit lipid peroxidation and ferroptosis by reducing lipid free radicals. As an NADPH-dependent oxidoreductase of CoQ10, the N-terminal myristoylation of FSP1 can promote its targeting of the plasma membrane and mediate NAD(P)H-dependent CoQ10 reduction, thereby inhibiting the activity of CoQ10. Therefore, the FSP1/CoQ_10_/NADPH system inhibits lipid peroxidation and ferroptosis by blocking the formation of the MDM2-MDMX complex. Overexpressing FSP1 in cells lacking GPX4 expression significantly reduced specific phospholipid peroxidation products compared to control cells. Moreover, cells lacking FSP1 expression have increased sensitivity to ferroptosis inducers, including the GPX4 inhibitor ML162 and the system Xc^-^ inhibitor erastin.

FSP1 overexpression can also induce cell death and reduce the sensitivity of cells to ferroptosis. Clinical studies found that the FSP1 level was significantly related to the lower overall survival rate of many cancer patients; thus, it can be used as a prognostic marker. Additionally, in clinical treatment, GPX4 upregulation promotes ferroptosis of tumor cells and may fail to achieve the desired effect because of the activation of the compensatory mechanism of the FSP1 system. Therefore, increasing the expression of GPX4 and FSP1 can synergistically promote the ferroptosis of tumor cells. In short, subcellular localization and expression levels and the activity of NADPH oxidoreductase are the key factors by which FSP1 inhibits cell ferroptosis; however, the underlying molecular mechanism has not yet reached a consensus. At present, studies on the mechanism by which FSP1 regulates ferroptosis are still in the initial stage, so comprehensive and in-depth exploration* is needed.*

### GCH1/BH4/DHFR system

Recently, genome-wide CRISPR/cas9 screening technology identified a GPX4-independent novel ferroptosis inhibitory mechanism called the GCH1/BH4/DHFR system^31^, ^32^. GCH1 has been regarded as the rate-limiting enzyme for catalyzing BH4 biosynthesis and is also a potential ferroptosis antagonist. BH4 is a free radical capture antioxidant; however, its recycling requires the participation of DHFR. Therefore, blocking DHFR can cooperate with GPX4 inhibitors to induce ferroptosis. Studies have proven that overexpression of GCH1 could promote the biosynthesis of BH4 and reduce CoQ10 levels, thereby hindering lipid peroxidation^31^, ^32^. In addition, it was also found that GCH1 could selectively protect phospholipids with two PUFA tails from lipid peroxidation degradation^33^. Similar to FSP1, GCH1 expression is significantly correlated with ferroptosis resistance, and GCH1 inhibition can enhance erastin-induced ferroptosis by promoting ferritinophagy, suggesting that the GCH1/BH4/DHFR system might serve as a novel ferroptosis defensive mechanism and a promising therapeutic target^34^. Although the role of GCH1 in protecting tissues and organs from ferroptosis has been gradually revealed, its specific mechanism in regulating ferroptosis remains to be further clarified.

### Dihydroorotate dehydrogenase (DHODH)/CoQH_2_ system

Mitochondria are the major site of aerobic respiration and the main source of ROS in cells, and mitochondrial ROS are crucial in initiating lipid peroxidation and ferroptosis^35^. Dihydroorotic acid dehydrogenase (DHODH), located on the mitochondrial inner membrane, is in charge of catalyzing pyrimidine nucleotide synthesis and can reduce ubiquinone (CoQ) to ubiquinol (CoQH_2_). A recent study reported that DHODH plays a key role in preventing the occurrence and development of ferroptosis^36^. Evidence suggests that when GPX4 is rapidly inactivated, cells can neutralize lipid peroxidation and prevent mitochondria injury-mediated ferroptosis by upregulating the expression levels of DHODH and CoQH_2_. Notably, mitochondrial GPX4 and DHODH can jointly inhibit lipid peroxidation through mutual compensation^37^.

The ferroptosis defense system is mainly divided into two parts: the GPX4 system and the CoQH2 system; the former includes cytosolic GPX4 and mitochondrial GPX4, and the latter includes FSP1 and mitochondrial DHODH. This classification of ferroptosis defense may be beneficial to reduce the accumulation of lipid peroxides in mitochondria and cope with the double membrane structure of mitochondria^38^. Therefore, DHODH/CoQH_2_ and mitochondrial GPX4 constitute two major ferroptosis defense systems to eliminate cell damage or death caused by the accumulation of mitochondrial lipid peroxides, and preclinical studies have shown that targeting DHODH can promote ferroptosis^36^. Since ferroptosis is activated in tumor cells, targeting DHODH to induce ferroptosis and inhibit tumor growth may provide a new target for cancer treatment.

### Transcription regulators

Ferroptosis is a complex regulatory network involving multiple regulatory factors, and various transcription factors can control the expression of downstream genes related to ferroptosis. Many studies have shown that transcription factors such as p53, Nrf2, ATF3, ATF4, YAP1, TAZ, SP1, HIF1A, BACH1, TFEB, JUN and HNF4A play key roles in regulating ferroptosis through transcriptional- or nontranscriptional-dependent mechanisms. Herein, we described the mechanisms of several key transcription factors, such as p53, Nrf2, ATF4, YAP1, and TAZ, in regulating ferroptosis in kidney diseases (Figure 1D). Among these, p53 exhibits a double-edged sword effect via distinct molecular mechanisms in the regulation of ferroptosis, which is very interesting^39^. For one thing, p53 as the upstream transcription factor, it can promote ferroptosis by inhibiting the expression of ALOX12 and SLC7A11, or by enhancing that of SAT1 (spermidine/spermine N1-acetyltransferase 1) and GLS2 (glutaminase 2). In addition, p53 displays the potential to inhibit ferroptosis by blocking the activity of dipeptidyl-peptidase-4 (DDP4) or by inducing the expression of CDKN1A/p21 (cyclin-dependent kinase inhibitor 1 A). Nuclear factor E2-related factor 2 (Nrf2) is an important redox-sensitive transcription factor that plays a crucial role in maintaining cellular redox balance by interacting with antioxidant response elements (ARE) and Kelch-like ECH-associated protein 1 (Keap1). A growing body of evidence has reported that Nrf2 participates in regulating ferroptosis by promoting the expression of downstream genes^40^, such as FTH1, SLC7A11, and HMOX1, most of which exert a negative regulatory effect against ferroptosis by promoting the synthesis of GSH, reducing ROS production, and upregulating NADPH expression. YAP/TAZ, as a transcriptional coactivator, is an important effector molecule in the Hippo pathway. Studies have indicated that YAP/TAZ regulates ferroptosis in various ways by transcriptionally inhibiting the expression of downstream genes such as SLC7A11, TFR-1 and ACSL4^41^. ATF4 is a key regulator of oxidative homeostasis and the cell cycle. New research has suggested that ATF4 suppresses ferroptosis by transcriptionally upregulating the expression of SLC7A11 and HSPA5^42^. On balance, various transcription regulators seem to crosstalk with each other to mediate cell ferroptosis.

## The emerging roles of organelles in ferroptosis

**Figure 2:**
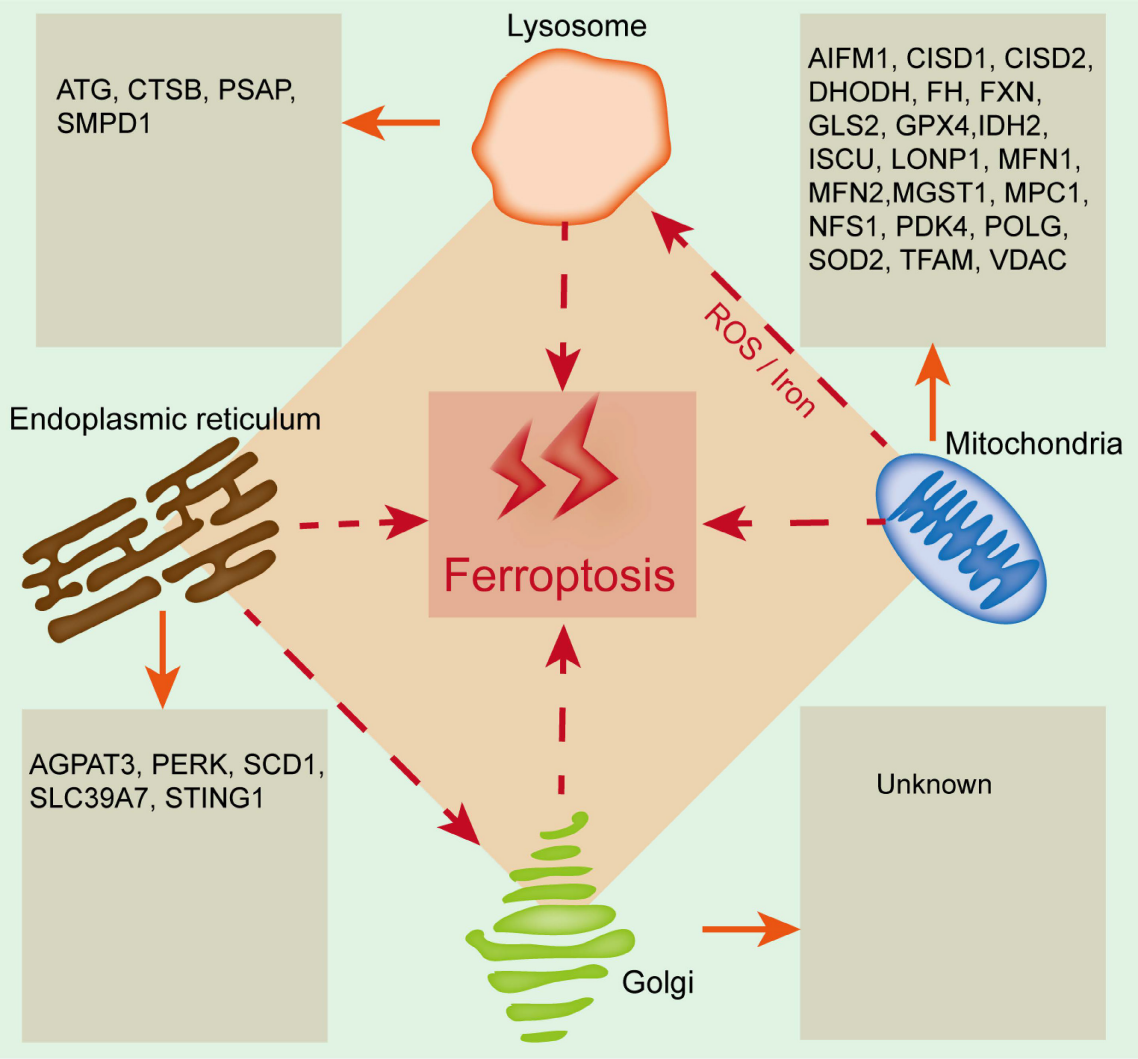
** The emerging roles of organelles in ferroptosis.** Widely studied organelles such as mitochondria, lysosomes, the endoplasmic reticulum and the Golgi apparatus have been confirmed to be involved in the regulation of ferroptosis through a variety of mechanisms, and oxidative damage to the lipid bilayer membrane of organelles is the core mechanism. CTSB, Cathepsin B; PSAP, Prosaposin; SMPD1, Sphingomyelin phosphodiesterase 1; AIFM1, Apoptosis inducing factor mitochondria-associated 1; CISD1, CDGSH iron sulfur domain 1; CISD2, CDGSH iron sulfur domain 2; DHODH, Dihydroorotate dehydrogenase; FH, Familial hypercholesterolemia; FXN, Frataxin; GLS2, Glutamine synthase 2; IDH2, Isocitrate dehydrogenase 2; ISCU, Iron-sulfur cluster scaffold protein; LONP1, Lon protease 1; MFN1, Mitofusin-1; MFN2, Mitofusin-2; MGST1, Microsomal glutathione-S-transferase 1; MPC1, Mitochondrial pyruvate carrier 1; NFS1, Cysteine desulfurase; PDK4, Pyruvate dehydrogenase kinase 4; POLG, DNA polymerase gamma; SOD2, Superoxide dismutase 2; TFAM, Mitochondrial transcription factor A; VDAC, Voltage-dependent anion channel; AGPAT3, 1-acylglycerol-3-phosphate O-acyltransferase 3; PERK, RNA-dependent protein kinase (PKR)-like ER kinase; SCD1, Stearyl-coenzyme A desaturase 1; SLC39A7, Solute carrier family 39 member 7; STING1, Stimulator of interferon response cGAMP interactor 1.

Organelles play important roles in maintaining cellular homeostasis, and their damage or dysfunction promotes cell death and induces the development of many diseases, including kidney diseases. Ferroptosis is closely related to and regulated by organelle stress. Mitochondria, lysosomes, the endoplasmic reticulum and the Golgi apparatus are involved in regulating ferroptosis, and the relevant regulatory mechanism is due to the lipid bilayer membrane structure of organelles, which is related to lipid metabolism (**Figure 2**). Therefore, exploring the relationship between ferroptosis and organelles is conducive to understanding ferroptosis from a new perspective, which will provide new ideas for basic and clinical studies on the prevention and treatment of kidney disease.

### Mitochondria and ferroptosis

Mitochondria have long been considered the key organelle for the integration of cell death and survival signals and are closely related to a variety of programmed cell death processes^43^. Mitochondria may be the main site for free iron to participate in lipid peroxidation by binding heme. Additionally, glutamine metabolism in the mitochondrial tricarboxylic acid (TCA) cycle plays a key role in ferroptosis, and erastin-induced ferroptosis can be blocked by inhibiting glutamine decomposition or intracellular glutamine deficiency^44^.

At present, the role of mitochondria in ferroptosis remains controversial. Studies have shown that inhibition of the mitochondrial TCA cycle or functional mitochondrial electron transport chain (ETC) can alleviate ferroptosis caused by cysteine deprivation. In addition, lipid peroxidation of the mitochondrial membrane is also an important source of lipid peroxides during ferroptosis. Under the condition of cysteine deficiency, mitochondrial TCA is enhanced, and lipid ROS production is increased, which promote the hyperpolarization and final collapse of the mitochondrial membrane, thereby resulting in ferroptosis^45^. Krainz et al. confirmed that the mitochondrial ROS-targeted scavenger XJB-5-131 effectively inhibited ferroptosis of human fibrosarcoma HTl080 cells^46^. Gao et al. found that erastin restricted the uptake and synthesis of cysteine in HTl080 cells by inhibiting system XC^-^, which confirmed that mitochondria are indispensable for cysteine deficiency-induced ferroptosis^38^. Su et al. reported that mitochondrial oxidative stress-induced ferroptosis enhanced the progression of acute renal tubular epithelial cell injury^47^. To date, a variety of mitochondria-resident proteins, such as CDGSH iron sulfur domain 1 (CISD1), FUN14 domain containing 1 (FUNDC1) and acyl-CoA synthetase family member 2 (ACSF2), have been confirmed to participate in regulating ferroptosis and kidney diseases^48^, ^49^. All the above findings clearly support the close link between mitochondria and ferroptosis.

### Endoplasmic reticulum (ER) and ferroptosis

During the process of pathological stimulation, the accumulation of misfolded and unfolded proteins leads to ER dysfunction and triggers ER stress (ERS)^50^. Ferroptosis is accompanied by ERS, but the specific role of the ER in ferroptosis is still unclear. Evidence has shown that ERS induces the upregulation of activating transcription factor 3 (ATF3), which further promotes iron overload and aggravates oxidative stress, ultimately resulting in glioma cell ferroptosis^51^; ferroptosis inhibition can reduce ERS and alleviate myocardial ischemia‒reperfusion injury in diabetes^52^. Zip transporter ZIP7 (SLC39A7) is a newly discovered genetic determinant of ferroptosis that can trigger ERS and inhibit ferroptosis^53^. Activating transcription factor 4 (ATF4) is a major signal transduction pathway for ERS to participate in regulating ferroptosis, which can increase cation transport regulator-like protein 1 (CHAC1) and promote GSH degradation, thus triggering ferroptosis^54^.

In addition, ferroptosis has a common regulatory pathway with other types of cell death^55^. Ferroptosis inducers, such as erastin and artesunate, can induce ERS, activate the eIF2α/ATF4/CHOP signaling pathway, upregulate p53 and then promote apoptosis, which indicates that ERS may be the common pathway of ferroptosis and apoptosis^56^. Recent studies have suggested that lipid ROS mainly accumulate in the mitochondrial matrix and ER in RSL3-treated mouse embryonic fibroblast cells^57^. The mitochondria-associated endoplasmic reticulum membrane (MAM), a key important membrane contact area between the mitochondria and ER, provides a platform for enhancing communication between the ER and mitochondria^58^. A growing body of evidence has supported that MAM dysfunction promotes the initiation and development of diseases by regulating ferroptosis. Li et al. demonstrated that MAM dysfunction induced cell ferroptosis in arsenic-induced acute lung injury mice by reducing MFN2 expression and inhibiting the interaction between mitochondria and ER^59^. Ta et al. found that MAM-resident protein FUNDC2 participated in ferroptosis by regulating mitochondrial GSH levels through the FUNDC2-SLC25A11 axis in doxorubicin-induced cardiomyopathy^60^. Wang et al. confirmed that the MAM-resident protein ACSL4 promoted ferroptosis-mediated acute kidney injury^61^. Based on the study of MAMs, it is suggested that the regulation of the MAM-mediated multiorganelle communication network plays a vital role in regulating ferroptosis in kidney diseases. Notably, crosstalk between ERS and ferroptosis might be a therapeutic target for the treatment of kidney diseases. Liang et al. found that ERS inhibition could alleviate ferroptosis and improve ischemia‒reperfusion-induced AKI^62^. Zhao et al. reported that ERS is involved in cadmium-induced ferroptosis of renal tubular epithelial cells^63^. Therefore, clarifying the crosstalk between ERS and ferroptosis may provide new insight into the pathogenesis of kidney diseases.

### Golgi apparatus and ferroptosis

The Golgi apparatus is the key protein processing factory in eukaryotic cells. As an important membranous organelle, the Golgi apparatus can process, modify, classify and package proteins synthesized by the ER and further transport them to specific parts of cells. Inhibiting Golgi apparatus homeostasis induces autophagy, apoptosis, and ferroptosis^64^. It has been demonstrated that Golgi apparatus stress induces the accumulation of lipid peroxides and GSH consumption. Recent evidence suggests that Golgi apparatus inhibitors such as BFA, GCA or AMF-26 can induce ferroptosis by decreasing GSH levels and enhancing lipid peroxidation in cancer cells. Moreover, ferroptosis inhibitors can effectively suppress Golgi apparatus stress by affecting its morphology and function, which further promotes the recovery of Golgi apparatus homeostasis^65^.

### Lysosomes and ferroptosis

Lysosomes are organelles rich in multiple acid hydrolases, which are involved in regulating cell metabolism, signal transduction, cell death and other cellular processes^66^. Recent studies have shown that lysosomes can affect the initiation and development of ferroptosis by reducing the transport of transferrin or the phagocytosis and degradation of ferritin^64^. Inhibition of lysosomal enzyme activity could block GSH depletion, reduce ROS accumulation, and prevent erastin-induced ferroptosis^67^. Mancias et al. revealed that the lysosomal inhibitor CQ promoted the binding of nuclear receptor coactivator 4 (NCOA4) to ferritin in MCF-7 cells and further mediated ferrinophagy, indicating the importance of NCOA4 in iron homeostasis and ferroptosis^68^. A recent study found the colocalization of lipid peroxides and lysosomes, and lysosomal inhibitors could suppress ferroptosis by reducing the intracellular transport of transferrin or blocking autophagic degradation of ferritin^69^. In addition, Yang et al. revealed that both the lysosomal inhibitor CQ and autophagy inhibitor spautin-1 could inhibit RSL3-induced ferroptosis in human non-small cell lung carcinoma cells^70^. These studies revealed the key role of lysosomes in ferroptosis. Since lysosomes can directly participate in the degradation of other organelles, the crosstalk between lysosomes and other organelles in regulating ferroptosis needs to be further explored.

## Pharmacological progress of ferroptosis

As the role of ferroptosis in the occurrence and development of various kidney diseases have been gradually recognized, alleviating the progress of diseases via the inhibition or activation of ferroptosis has become a hot research topic. At present, a variety of natural and synthetic drugs associated with ferroptosis have been found, including inducers and inhibitors. For nonneoplastic kidney diseases, renal function can be improved by the application of ferroptosis inhibitors, such as lipid peroxidation pathway inhibitors, iron homeostasis regulators, and ROS generation inhibitors. However, in renal cell carcinoma, ferroptosis inducers can be used to prevent the development of cancer. The inhibitors and inducers that modulate ferroptosis are described in **Table 2**.

## The key role of ferroptosis in kidney diseases

**Figure 3:**
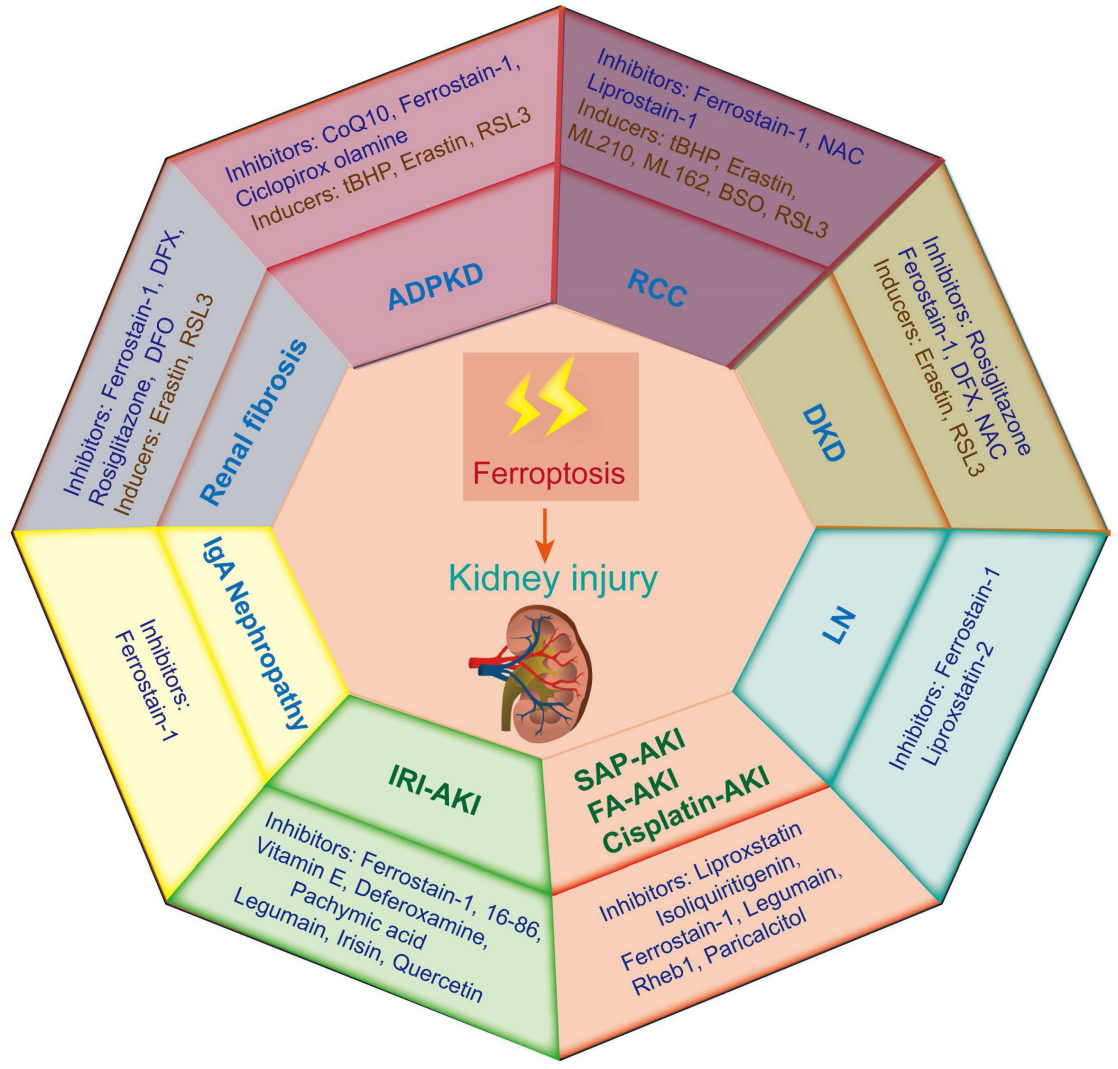
** Ferroptosis-related inducers and inhibitors in various kidney diseases.** Ferroptosis occurs in various animal models of kidney diseases, including renal cell carcinoma (RCC), acute kidney injury (AKI), diabetic kidney disease (DKD), autosomal dominant polycystic kidney disease (ADPKD), renal fibrosis, lupus nephritis (LN) and IgA nephropathy. IRI-AKI, ischemia‒reperfusion injury induced AKI; FA-AKI, folic acid induced AKI.

Ferroptosis-induced tissue dysfunction has long been considered a noticeable critical factor in the progression of a variety of kidney diseases^71^. Therefore, this study aims to summarize the recent progress on the role of ferroptosis in different kidney diseases to provide strong support for the treatment and prevention of these diseases (**Figure 3**).

### Renal cell carcinoma (RCC)

Renal cell carcinoma (RCC) is a frequent human malignancy and accounts for approximately 90% of malignant renal tumors. Epidemiological statistics show that the incidence rate of RCC is significantly increased and ranks second in urogenital system tumors around the world^72^. As the most common pathological type of RCC, clear cell renal cell cancer (ccRCC) is characterized by abnormal lipid and glycogen accumulation and is ineffective for extensive anticancer treatment. Currently, the clinical prognosis of ccRCC is still unclear; therefore, it is urgent to explore new therapeutic targets^73^. Much evidence has proven that ccRCC is sensitive to ferroptosis, and inducing ferroptosis may be a new direction for the treatment of ccRCC^74^. A sensitivity profiling of 117 kinds of cancer cell lines to erastin-induced ferroptosis revealed that ccRCC was particularly sensitive to GPX4-mediated ferroptosis, which first suggested that ferroptosis might be a potential target for RCC^75^.

The Hippo pathway plays a key role in cell density and confluency, and high cell density leads to ferroptosis. TAZ, a key transcription regulator in the Hippo pathway, is highly expressed in ccRCC and closely associated with cell density-influenced ferroptosis susceptibility^76^. Zou et al. found that GPX4 inhibitors effectively destroyed renal clear carcinoma cells, and their further investigation demonstrated that overexpression of hypoxia-inducible factor 2α (HIF-2α) could significantly enhance the ferroptosis sensitivity of ccRCC by enriching PUFAs 77. Von hippel-lindau (*VHL*) is a major tumor suppressor gene, and overexpression of VHL in ccRCC could effectively inhibit ferroptosis by reducing lipid peroxide accumulation, indicating that *VHL*-induced ferroptosis might be a promising target for ccRCC treatment^78^. Iron sulfur cluster assembly 2 (ISCA2) is an essential part of the mitochondrial iron sulfur cluster assembly complex, and decreased ISCA2 levels are associated with VHL loss, HIF-2α downregulation and enhanced ferroptosis sensitivity. Therefore, targeting ISCA2 is a potential strategy to induce ferroptosis in ccRCC^79^.

ACSL4 is a ferroptosis indicator that is downregulated in ccRCC and is considered a valuable biomarker for ccRCC treatment^80^. In addition, the genes *KLF2*^81^, *metallothionein 1 G (MT1G)*^82^ and *KDM5C^83^* also play positive roles in preventing or delaying ccRCC development by promoting GSH metabolism and inhibiting lipid peroxidation. Ferrinophagy is a type of cell-selective autophagy mediated by NCOA4, which can degrade ferritin autophages, leading to the release of ferritin-bound iron into free iron^84^. NCOA4 was downregulated in patients with ccRCC, and targeting NCOA4 to promote ferroptosis might be an effective therapeutic strategy^85^. Massive accumulation of intracellular fumarate caused by the inactivation of fumarate hydratase decreases GPX4 activity through posttranslational modification, which further increases the sensitivity of ccRCC to ferroptosis^86^.

Recently, with the rapid study of traditional Chinese medicine, a variety of Chinese herb components have been found to prevent the progression of ccRCC by inducing ferroptosis in drug-resistant renal carcinoma cells, such as artesunate^87^, icariside II^88^, erianin^89^, everolimus^90^ and lycorine^91^. Taken together, we easily believe that a profound study of ferroptosis may provide new ideas for ccRCC treatment.

### Acute kidney injury (AKI)

AKI is characterized by a rapid decline in renal function and is caused by various pathological factors, such as rhabdomyolysis (RM), oxidative stress, inflammation, hypoxia, infection, ischemia‒reperfusion injury (IRI), sepsis, and nephrotoxic drugs^92^. Previous studies found that the development of AKI promotes the potential risk of patients developing CKD and ESRD^93^. Therefore, it is important to seek potential targets for the treatment of AKI. The pathogenesis of AKI is very complex, and lipid peroxidation-dependent ferroptosis has been considered one of the key mediators of renal damage^10^. Many studies have demonstrated that ferroptosis is a potential target for AKI treatment, and ferroptosis inhibitors have a beneficial effect on AKI.

RM-induced renal failure accounts for 15% of AKI, and myoglobin (Mb) accumulation in the kidney is the core mechanism^94^. Recent findings showed that renal tubular epithelial cell ferroptosis caused by excessive iron produced by Mb metabolism may be a major mechanism in rhabdomyolysis-induced renal injury^95^. Free Fe^2+^ released from Mb degradation catalyzes the production of lipid peroxides through the Fenton reaction, which eventually induces ferroptosis and AKI. It was reported that ferroptosis was involved in the development of RM-induced AKI, and desferrilamine or curcumin could effectively alleviate kidney damage^96^, ^97^.

Since the kidney is highly sensitive to IRI, IRI is considered one of the major triggers for AKI and leads to renal tubular cell death and progressive kidney tissue damage^98^. IRI-induced AKI (IRI-AKI) was initially thought to be mainly caused by oxidative stress and inflammation; however, growing evidence has demonstrated that lipid peroxidation-dependent ferroptosis plays a key role in IRI-AKI development^99^. Studies have confirmed that activation of the GSH/GPX4 axis or knockout of ACSL4 could effectively inhibit tubular cell ferroptosis in an IRI-AKI mouse model^61^, ^100^. A recent study reported that conditional knockout of FSP1 or GPX4 increased the sensitivity of cell ferroptosis in an IRI-AKI mouse model^101^. Treatment with ferroptosis inhibitors, including XJB-5-131, ferrostatin-1, mitoglitazone, cyanidin-3-glucoside and liproxstatin-1, was proven to improve functional acute renal failure in an IRI-AKI mouse model^102^-^105^. Additionally, a variety of Chinese herb components could also effectively alleviate kidney damage in an IRI-AKI mouse model by inhibiting ferroptosis^106^-^108^.

Sepsis is another major cause of AKI, and further exploration of its mechanism will provide more possibilities for the prevention and treatment of SA-AKI. A recent study found that renal tubular epithelial cell ferroptosis occurred in SA-AKI mice, and Maresin conjugates in tissue regeneration 1 (MCTR1) suppressed ferroptosis in LPS-induced HK-2 cells and SA-AKI mice by activating Nrf2^109^. Guo et al. found that ginsenoside Rg1 could inhibit ferroptosis of renal tubular epithelial cells in SA-AKI by regulating the FSP1-CoQ10-NAD(P)H signaling pathway^110^. Meanwhile, Zhang et al. revealed that the noncoding RNA *miR-124-3p*.1 could regulate the LPCAT3-mediated ferroptosis pathway in SA-AKI, thereby indicating the importance of ferroptosis in SA-AKI.

AKI induced by nephrotoxic drugs, such as cisplatin and folic acid (FA), is also closely related to ferroptosis. Guo et al.^111^ and Martin-Sanchez et al.^112^ confirmed that iron overload and lipid peroxidation existed in an FA-induced AKI (FA-AKI) mouse model, and ferroptosis inhibition could effectively improve renal function and ameliorate kidney damage in FA-AKI. As a widely used chemotherapy drug, cisplatin has certain nephrotoxicity, which can lead to renal injury^113^.

Multiple studies have shown that cisplatin can promote renal tubular cell ferroptosis and enhance kidney damage in cisplatin-induced AKI (CI-AKI) mice, and inhibiting ferroptosis significantly reduces the levels of urea nitrogen and blood creatinine and further alleviates oxidative damage and fibrosis in kidney tissues^10^. Ferrostatin-1 or polydatin could inhibit ferroptosis in CI-AKI mice by regulating the GSH/GPX4 signaling pathway^114^. In a CI-AKI mouse model, selenium nanoparticle administration effectively inhibited ferroptosis by decreasing lipid peroxide accumulation and increasing GPX4 activity^115^. In addition, Hu et al. found that the activation of the vitamin D receptor could inhibit ferroptosis by upregulating GPX4 expression, thereby improving renal function and alleviating kidney injury in CI-AKI mice^116^. Xu et al. confirmed that dihydromyricetin could improve kidney injury in CI-AKI by reducing oxidative stress, inflammation and ferroptosis^117^. A recent study by Hu et al. demonstrated that leonurine ameliorated ferroptosis in CI-AKI by activating and upregulating the expression of Nrf2^118^. Therefore, ferroptosis plays a key role in AKI, and inhibiting ferroptosis may be an effective strategy to treat AKI.

### Diabetic kidney disease (DKD)

DKD is a common and frequent microvascular complication of diabetes and has become the predominant cause of end-stage renal disease (ESRD)^119^. The major pathological manifestations of DKD are glomerulosclerosis, glomerular basement membrane thickening, extracellular matrix accumulation, tubulointerstitial injury and fibrosis, which eventually lead to renal failure^120^. The pathogenesis of DKD involves many factors, including glucose metabolic dysfunction, oxidative stress, inflammation, and hemodynamic changes. It has been reported that renal cell death is closely related to the development of DKD^121^. Ferroptosis is an important driver of DKD, and iron-dependent lipid peroxidation has been widely found in DKD patients^122^ and DKD animal models, such as *DBA/2J* mice^123^, *db/db* mice^124^, and streptozotocin (STZ)-induced mouse^125^ or rat^126^ models. Kim et al. found that the expression levels of SLC7A11 and GPX4 in the renal tubules of DKD patients were significantly downregulated compared with those in non-DKD patients^122^. Upregulating the expression of Nrf2 or ferrostatin-1 treatment could significantly increase GPX4 activity, thereby inhibiting renal tubular epithelial cell ferroptosis and alleviating kidney injury in *DBA/2J* mice^123^. With the in-depth study of DKD and ferroptosis, researchers found that peroxiredoxin 6 (Prdx6)^127^ and ZRT/IRT-like protein 14 (ZIP14)^126^ played key roles in DKD, and upregulating Prdx6 or downregulating ZIP14 could effectively inhibit ferroptosis and delay DKD progression. Additionally, recent studies have shown that small molecular reagents, including N-acetylcysteine (NAC), dapagliflozin and salusin‑β, can effectively improve renal function and alleviate kidney injury by suppressing ferroptosis. It has also been reported that many Chinese herbal components, such as glabridin^128^, umbelliferone^129^, schisandrin A^130^, ginkgolide B^131^, calycosin^132^, quercetin^133^, vitexin^134^, dapagliflozin^135^ and platycodin D^136^, can effectively inhibit the oxidative stress and ferroptosis of renal tubular epithelial cells and thus exert a positive effect on improving renal function in DKD.

Interestingly, studies on noncoding RNAs and DKD found that circ ASAP2^137^ and mmu_circRNA_0000309^138^ could alleviate diabetic kidney injury by targeting ferroptosis. Obviously, a growing body of evidence supports the key role of ferroptosis in the occurrence and development of DKD; however, direct evidence on ferroptosis and DKD progression is still lacking. Therefore, further investigation of the role and mechanism of ferroptosis in DKD can provide new ideas for the clinical diagnosis and treatment of DKD^139^.

### Autosomal dominant polycystic kidney disease (ADPKD)

ADPKD, caused by mutations in *PKD1* or *PKD2*, is one of the most common dominant hereditary kidney diseases and is characterized by the progressive development of bilateral renal epithelial cysts^140^. The formation and enlargement of cysts can destroy the function of the renal parenchyma, further promoting ADPKD progression to CKD and even ESRD. Therefore, suppressing the occurrence and progression of renal cysts is of great significance for the treatment of ADPKD.

Oxidative stress, inflammation, hypoxia and cell death play key roles in cyst formation and progression in ADPKD. Interestingly, several studies strongly supported that ferroptosis was involved in ADPKD development^141^. TMEM16A is a chloride bicarbonate transmembrane channel that is closely associated with renal cyst growth in ADPKD. Schreiber et al. found that lipid peroxidation promoted the activation of TMEM16A and cyst enlargement in kidney tissues of ADPKD patients and a mouse model. However, ferrostatin-1 treatment could effectively suppress TMEM16A activation and renal cyst growth, which supported that ferroptosis is a possible factor during ADPKD progression^142^. A recent investigation in *PKD1* mutant kidney cells and mice showed that the expression levels of system Xc^-^, ferroportin and GPX4 were significantly decreased, accompanied by increased levels of TFR1 and DMT1. In addition, both ADPKD cell and mouse models displayed high iron content, low GPX4 activity, increased lipid peroxide accumulation, and a tendency toward ferroptosis; ferroptosis inhibitors effectively suppressed ferroptotic cell death and cyst growth in ADPKD1 mutant mice^143^. These findings implied that ferroptosis is a key contributor to ADPKD progression, and targeting ferroptosis may be a promising strategy for the treatment of ADPKD.

### Renal fibrosis

When the kidney is stimulated by multiple pathogenic factors and with the inflow of fibrosis factors and cytokines, intrinsic renal cells undergo transdifferentiation, thereby inducing massive proliferation of fibroblasts and myofibroblasts and excessive accumulation of extracellular matrix, which further leads to glomerulosclerosis and tubular fibrosis and ultimately results in the loss of renal function^144^. There is a general belief that renal fibrosis is the major risk factor in the progression of chronic kidney disease (CKD) to ESRD^145^. In the early stage of CKD, renal tubular epithelial cell injury triggers a series of immune responses, and excessive inflammatory responses further aggravate cell damage and death. However, cell death also has a strong proinflammatory effect, which can worsen the damage to renal tubules through feedback regulation, and persistent inflammation and damage eventually result in renal tubulointerstitial fibrosis^146^.

Recent studies have suggested that ferroptosis plays a key role in the progression of renal tubular injury to renal fibrosis^147^. To date, ferroptosis has been found in a variety of renal fibrosis animal models, such as the unilateral ureteral obstruction (UUO) mouse model^148^-^150^ and the 5/6 nephrectomy-induced CKD rat model^151^. A recent study by Gong et al. found repressor element 1-silencing transcription factor induced the transition of renal injury to renal fibrosis through regulating ferroptosis of renal tubular epithelial cells^152^. Studies have found that ferroptosis inducers decrease GPX4 activity and enhance intracellular lipid peroxidation, further exacerbating the progression of renal injury and fibrosis. However, pretreatment with ferroptosis inhibitors, such as liproxstatin-1^148^, tocilizumab^149^, rhein^153^, formononetin^154^ and tectorigenin^150^, could prevent the development of renal fibrosis by inhibiting ferroptosis-related lipid peroxidation and GSH consumption.

A recent study by Li et al. also reported that roxadustat (FG-4592) pretreatment ameliorated renal injury and renal fibrosis by inhibiting renal tubular epithelial cell ferroptosis via the Akt/GSK3β/Nrf2 signaling pathway in folic acid-induced AKI^155^. Substantially, recent evidence suggests that ferroptosis plays a core role in the progression of renal fibrosis and that suppressing ferroptosis benefits tissue remodeling and fibrosis regression. Although many studies on ferroptosis and renal fibrosis have been reported, more investigation is needed to clarify whether ferroptosis can prevent and delay renal fibrosis by directly reducing the inflammatory reaction or has a direct impact on renal fibrosis.

### Lupus nephritis

Lupus nephritis (LN) is an immune injury caused by systemic lupus erythematosus involving different pathological types of kidneys. The pathogenesis of LN involves multiple factors, including environmental factors, genetic factors, hormone regulation, immune complex clearance, and cell metabolism^156^; however, its specific pathogenesis is still unclear. Therefore, it is of great significance for future clinical diagnosis and treatment to deeply investigate the pathogenesis and explore specific therapeutic targets.

Recent findings revealed a close linkage between ferroptosis and LN^11^. Wang et al. confirmed that lipid peroxide accumulation with ferroptosis-related differentially expressed genes was aberrantly activated in both glomeruli and tubulointerstitium in LN^157^. A recent study reported a serious increase in iron overload and lipid peroxidation in the kidney tubules of lupus-prone nephritic mice and LN patients through proteomics and lipidomics-based approaches. Meanwhile, this study observed downregulation of GPX4 and upregulation of ACSL4 in the kidney tissue of LN mice. Furthermore, this study also confirmed that liproxstatin-2, a new ferroptosis inhibitor, possessed a prophylactic and therapeutic benefit in alleviating LN patient serum-induced renal tubular epithelial cell ferroptosis^158^. Taken together, these results strongly highlight that renal tubular epithelial cell ferroptosis might be a pathological feature in LN patients and mouse models, and ferroptosis inhibitors are considered potential novel adjunct therapeutics to treat LN.

### IgA nephropathy

IgA nephropathy is one of the most common primary glomerular diseases. Previous studies suggested that abnormal glycosylation of IgA1 is a key link in the progression of IgA nephropathy^159^; however, the newest study supported that ferroptosis was an important trigger for IgA nephropathy^160^. Wu et al. confirmed that the expression level of GPX4 in the kidney tissues of patients with IgA nephropathy was significantly reduced. They also found that Gd-IgA1 stimulation caused a decrease in the activity of mesangial cells, accompanied by iron overload, lipid peroxide accumulation, mitochondrial morphological abnormalities and other biochemical and pathological phenotypes closely related to ferroptosis^161^. Furthermore, treatment with the ferroptosis inhibitor Fer-1 significantly upregulated the expression of GPX4 and improved mitochondrial damage by reducing ROS and MDA levels, thereby inhibiting mesangial cell ferroptosis and delaying the development of IgA nephropathy^161^. Therefore, ferroptosis may be a potential target for the prevention and treatment of IgA nephropathy.

### Outlook

Ferroptosis is a kind of programmed death that can be regulated by various mechanisms and characterized by iron overload and lipid peroxidation. A growing body of evidence has proven that ferroptosis plays an important role in many chronic diseases^162^, such as heart diseases, neurodegenerative diseases and liver diseases, suggesting that it is an effective target for drug development and disease treatment. Although studies have confirmed that ferroptosis is closely related to the pathophysiological process of many kidney diseases, the molecular biological mechanism of ferroptosis involved in kidney injury is still lacking in-depth understanding. In this study, we aimed to explore the regulatory mechanism of ferroptosis, discuss the research progress of ferroptosis inhibitors or inducers, and prospect for its potential as a therapeutic target for kidney diseases.

Currently, the study of ferroptosis in kidney diseases is in the early stages, and no specific biomarkers have been determined^163^. Therefore, further investigation of the ferroptotic biomarkers has broad prospects for performing targeted therapies in kidney diseases. With advances of studies, many new regulators related to ferroptosis have been confirmed, such as ACSL4^61^, bone morphogenetic protein-7 (BMP7)^164^ and ZRT/IRT-like protein 14 (ZIP14)^126^, which will further promote the understanding of the regulatory mechanism of ferroptosis and provide promising directions for expanding novel drugs for the treatment of kidney diseases in the future.

At present, in view of extensive research on key mechanisms and regulators of ferroptosis, a large number of small molecule drugs have been found to induce or inhibit ferroptosis to alleviate renal tissue injury in various kidney diseases, such as RSL3, erastin, Fer-1, vitamin K and quercetin. However, these drugs have not been transformed into clinical applications to benefit patients with kidney diseases^165^. We believe that further studies can start from the following aspects: 1) further clarify the mechanism of ferroptosis and uncover more regulators for the important targets of ferroptosis; 2) continue to explore the relationship between the regulatory mechanism of ferroptosis and kidney-related diseases and study the role of ferroptosis in chronic kidney disease; 3) explore the regulation of ferroptosis mediated by epigenetic modification; and 4) transform small molecule drugs that can be used to regulate ferroptosis and use them in the clinic. This review systemically describes the mechanism of ferroptosis and its research advances in kidney diseases, thereby providing a solid foundation for the treatment, diagnosis and further clinical studies of kidney diseases.

Although many studies have reported the close relationship between ferroptosis and kidney diseases, compared with the extensive evidence on ferroptosis in oncology, studies on ferroptosis in kidney diseases are still scarce, and the regulatory mechanisms, modulators, signaling pathways and drug targets still need to be expanded. Currently, investigations on ferroptosis and kidney diseases are mainly focused on RCC and AKI; however, there are relatively few studies on other types of kidney diseases. Therefore, it is of great significance to conduct a profound study on the mechanism and role of ferroptosis in renal fibrosis, DKD, LN, ADPKD and IgA nephropathy. To date, ferroptosis-related inducers and inhibitors for the treatment of kidney injury have made great progress, but the vast majority of them remain in the animal and preclinical experimental stages. Therefore, it is worth looking forward to the clinical transformation and the development of new precision drugs (such as nano-targeted drugs). In summary, although this study aims to comprehensively review the pathogenesis of ferroptosis in different kidney diseases, we still believe it has some shortcomings, such as the limitations of research perspectives. Notably, with in-depth studies on ferroptosis in renal diseases and the elucidation of its mechanism, we believe it will provide new intervention targets for the prevention and treatment of various kidney diseases in the future, and we are also planning to regularly conduct systemic summaries of this field of research.

## Figures and Tables

**Table 1:** Comparison of different types of cell death

Types of cell death	Biochemical features	Morphological features	Immune features	Regulatory Pathways	Key genes
**Ferroptosis**	Inhibition of Xc^-^ and GPX4, reduced GSH, Iron accumulation and lipid peroxidation	Small mitochondria with condensed mitochondrial membrane densities, reduction or vanishing of mitochondria crista, as well as outer mitochondrial membrane rupture.	Pro-inflammatory	System Xc^-^/GPX4, MVA, HSF1/HSPB1, p62/Keap1/Nrf2	*GPX4*, *SLC7A11*, *Nrf2*, *ACSL4*, *FSP1*
**Ferritinophagy**	Increased lysosomal activity, iron accumulation and lipid peroxidation	Mitochondria atrophy, chromatin concentration, organelle swelling, plasma membrane rupture, formation of double-membraned autolysosomes.	Pro-inflammatory	Iron homeostasis disorder, ROS, ATG12/ATG5/LC3	*NCOA4*, *ATG5*, *ATG7*, *LC3B*, *FTH1*, *SOD2*, *SOX4*
**Cuproptosis**	Cu^2+^ overload triggers disruption of iron-sulfur cofactors; excessive ROS; Cu depedent-, mitochondria- induced cell death	Reduction of mitochondria volume and cristae; increased density of bilayer membrane structure.	Pro-inflammatory	TCA cycle, ROS, mitochondrial respiration, copper homeostasis disorder	*DLAT*, *PDHA1*, *PDHB*, *SLC25A3*, *FDX1*, *LIAS*, *HSP70*
**Apoptosis**	DNA fragmentation decreases the mitochondrial membrane potential	Plasma membrane blebbing, cellular and nuclear volume reduction, nuclear fragmentation.	Anti-inflammatory	Death receptor, mitochondria and endoplasmic reticulum pathway, caspase, p53, Bcl-2	*Caspases*, *Bcl-2*, *Bax*, *p53*, *Fas*
**Necroptosis**	Activation of kinases and drop in ATP levels	Plasma membrane rupture, organelle swelling, moderate chromatin condensation	Pro-inflammatory	TNF-α, TNFR1, TLR3, TRAIL, FasL, ROS, PKC/MAPK/AP-1	*LEF1*, *RIP1*, *RIP3*
**Pyroptosis**	Dependent on caspase-1 and proinflammatory cytokine releases	Karyopyknosis, cell edema and membrane rupture.	Pro-inflammatory	Caspase-1, NLRP3-mediated signaling pathway	*Caspase-1*, *IL-1β*, *IL-18*
**Autophagy**	Increased lysosomal activity	Formation of double-membraned autolysosomes.	Anti-inflammatory	PI3K/AKT/mTOR, MAPK/ERK1/2/ mTOR signaling pathway	*ATG5*, *ATG7*, *LC3*, *DRAM3*, *TFEB*

ACSL4: acyl-CoA synthetase long-chain family member 4, ALOX-15: arachidonate lipoxygenase 15, AP-1: activator protein-1, ATG5: autophagy-related 5, ATG7: autophagy-related 7, COQ10: coenzyme Q10, DRAM3: damage regulated autophagy modulator 3, FSP1: ferroptosis suppressor protein 1, GPX4: glutathione peroxidase 4, HSPB1: heat shock protein beta-1, Keap1: Keleh-like ECH-associated protein 1, MAPK: mitogen-activated protein kinase, MLKL: mixed lineage kinase domain like protein, mTOR: mammalian target of rapamycin, MVA: mevalonate, LC3: microtubule-associated protein 1 light chain 3, NCOA4: nuclear receptor coactivator 4, Nrf2: nuclear factor erythroid 2-related factor 2, PKC: protein kinase C, RIP: receptor-interacting serine/threonine kinase, ROS: reactive oxygen species, SAT1: spermidine/spermine N1-acetyltransferase 1, SLC7A11: solute carrier family 7 member 11, system Xc^-^: cysteine/glutamate transporter receptor, TFEB: transcription factor EB, TFR1: transferrin receptor 1, TNF-α: tumor necrosis factor α, TCA cycle: tricarboxylic acid cycle.

**Table 2:** Reagents that modulate ferroptosis

Classification	Reagents	Targets	Impact on ferroptosis
Ferroptosis inducers	Sulphasalazine	System Xc^-^	Prevents cystine import, causes GSH depletion
	Sorafenib	System Xc^-^	Prevents cystine import, causes GSH depletion
	Glutamate	System Xc^-^	Prevents cystine import, causes GSH depletion
	Erastin and its analogs	System Xc^-^, VDAC2/3	Prevents cystine import, causes GSH depletion
	RSL3	GPX4	Covalent inhibitor of GPX4 that causes accumulation of lipid hydroperoxides
	ML162	GPX4	Covalent inhibitor of GPX4 that causes accumulation of lipid hydroperoxides
	FINO_2_	GPX4	Covalent inhibitor of GPX4 that causes accumulation of lipid hydroperoxides
	FIN56	CoQ10 and GPX4	Depletes CoQ10 via SQS-mevalonate pathway and causes decrease in GPX4 protein abundance
	BSO, DPI2, cisplatin	GHS	GHS deletion
	Statins (e.g., cerivastatin, simvastatin)	HMGCR	Blocks CoQ10 biosynthesis
	Trigonelline, brusatol	Nrf2	Nrf2 inhibition
	Siramesine, lapatinib	Ferroportin, Transferrin	Upregulates cellular iron
	Brequinar	DHODH	Inhibits DHODH
	iFSP	FSP1	Inhibits FSP1
	BAY 87‐2243	Mitochondrial respiratory chain	Inhibits mitochondrial respiratory chain
	Andrographolide	Lipid peroxidation	Promotes lipid peroxidation
	Toosendanin	Lipid peroxidation	Promotes lipid peroxidation
	Gliotoxin	ROS, lipid peroxidation	Promotes lipid peroxidation
	Arsenic trioxide	ACSL4	ACSL4 activation
	Manganese	ROS, lipid peroxidation	Promotes lipid peroxidation
	Fatostatin	GPX4	Inhibits GPX4 expression
	Legumain	GPX4	Facilitates chaperone-mediated autophagy of GPX4
	Dihydroartemisinin (DHA)	Ferritin, iron overload	Increases cellular iron
	Artemisinins	Iron overload	Increases cellular iron
Ferroptosis inhibitors	Ferrostatin-1	Lipid peroxidation	Blocks lipid peroxidation
	Liproxstatin-1	Lipid peroxidation	Blocks lipid peroxidation
	Vitamin E	Lipid peroxidation	Blocks lipid peroxidation
	SRS 16-86, SRS 11-92	Lipid peroxidation	Blocks lipid peroxidation
	Troglitazone, Pioglitazone, Rosiglitazone	ACSL4	ACSL4 inactivation
	Deuterated polyunsaturated fatty acids (D-PUFAs)	Lipid peroxidation	Blocks lipid peroxidation
	XJB-5-131	Lipid peroxidation	Blocks lipid peroxidation
	Butylated hydroxytoluene, butylated hydroxyanisole	Lipid peroxidation	Blocks lipid peroxidation
	Ferrostatins, liproxstatins	Lipid peroxidation	Blocks lipid peroxidation
	CDC, PD-146176, AA-861, zileuton	Lipoxygenases	Blocks lipid peroxidation
	Selenium	Selenoproteins	Blocks lipid peroxidation
	Deferoxamine, cyclipirox, deferiprone	Intracellular iron	Decreases cellular iron
	Vildagliptin, alogliptin, and Linagliptin	DPP4	Blocks DPP4-mediated lipid peroxidation
	Irisin	GPX4	Upregulates GPX4
	Melatonin	System Xc^-^ and GPX4	Upregulates system Xc^-^ and GPX4
	Vitexin	System Xc^-^ and GPX4	Upregulates system Xc^-^ and GPX4
	Isoliquiritigenin	System Xc^-^ and GPX4	Upregulates system Xc^-^ and GPX4
	Vitamin A	Lipid peroxidation	Blocks lipid peroxidation
	Paricalcitol	GPX4	Upregulates GPX4
	Pachymic acid	Nrf2, GPX4, SCL7A11, and HO-1	Upregulates of Nrf2, GPX4, SCL7A11 and HO-1
	Rheb1	Maintains mitochondrial homeostasis	Maintains mitochondrial homeostasis
	Quercetin	System Xc^-^ and GPX4	Upregulates system Xc^-^ and GPX4
	Artesunate	Lipoxygenases	Blocks lipid peroxidation
	Curcumin	Lipoxygenases	Blocks lipid peroxidation
	Baicalein	Lipoxygenases	Blocks lipid peroxidation
	Nuciferine	Lipoxygenases	Blocks lipid peroxidation
	Vitamin K	Lipid peroxidation	Blocks lipid peroxidation
	Cardamonin	Lipid peroxidation	Blocks lipid peroxidation
	Proanthocyanidins	Lipid peroxidation	Blocks lipid peroxidation
	Uridine	Lipid peroxidation	Blocks lipid peroxidation
	NDGA	Lipoxygenases	Blocks lipid peroxidation

## References

[1] Jager KJ, Kovesdy C, Langham R, Rosenberg M, Jha V, Zoccali C (2019). A single number for advocacy and communication-worldwide more than 850 million individuals have kidney diseases. Kidney Int.

[2] Kovesdy CP (2022). Epidemiology of chronic kidney disease: an update 2022. Kidney Int Suppl (2011).

[3] Priante G, Gianesello L, Ceol M, Del Prete D, Anglani F (2019). Cell Death in the Kidney. Int J Mol Sci.

[4] Santagostino SF, Assenmacher CA, Tarrant JC, Adedeji AO, Radaelli E (2021). Mechanisms of Regulated Cell Death: Current Perspectives. Vet Pathol.

[5] Uysal E, Dokur M, Kucukdurmaz F, Altinay S, Polat S, Batcioglu K (2022). Targeting the PANoptosome with 3,4-Methylenedioxy-beta-Nitrostyrene, Reduces PANoptosis and Protects the Kidney against Renal Ischemia-Reperfusion Injury. J Invest Surg.

[6] Garg JP, Vucic D (2016). Targeting Cell Death Pathways for Therapeutic Intervention in Kidney Diseases. Semin Nephrol.

[7] Dixon SJ, Lemberg KM, Lamprecht MR, Skouta R, Zaitsev EM, Gleason CE (2012). Ferroptosis: an iron-dependent form of nonapoptotic cell death. Cell.

[8] Li J, Cao F, Yin HL, Huang ZJ, Lin ZT, Mao N (2020). Ferroptosis: past, present and future. Cell Death Dis.

[9] Zhang X, Li X (2022). Abnormal Iron and Lipid Metabolism Mediated Ferroptosis in Kidney Diseases and Its Therapeutic Potential. Metabolites.

[10] Feng Q, Yu X, Qiao Y, Pan S, Wang R, Zheng B (2022). Ferroptosis and Acute Kidney Injury (AKI): Molecular Mechanisms and Therapeutic Potentials. Front Pharmacol.

[11] Wlazlo E, Mehrad B, Morel L, Scindia Y (2021). Iron Metabolism: An Under Investigated Driver of Renal Pathology in Lupus Nephritis. Front Med (Lausanne).

[12] Wang S, Chen S, Ying Y, Ma X, Shen H, Li J (2021). Comprehensive Analysis of Ferroptosis Regulators With Regard to PD-L1 and Immune Infiltration in Clear Cell Renal Cell Carcinoma. Front Cell Dev Biol.

[13] Santana-Codina N, Gikandi A, Mancias JD (2021). The Role of NCOA4-Mediated Ferritinophagy in Ferroptosis. Adv Exp Med Biol.

[14] Wang Y, Zhang L, Zhou F (2022). Cuproptosis: a new form of programmed cell death. Cell Mol Immunol.

[15] Tang D, Chen X, Kang R, Kroemer G (2021). Ferroptosis: molecular mechanisms and health implications. Cell Res.

[16] Dixon SJ, Stockwell BR (2014). The role of iron and reactive oxygen species in cell death. Nat Chem Biol.

[17] Vogt AS, Arsiwala T, Mohsen M, Vogel M, Manolova V, Bachmann MF (2021). On Iron Metabolism and Its Regulation. Int J Mol Sci.

[18] Gaschler MM, Stockwell BR (2017). Lipid peroxidation in cell death. Biochem Biophys Res Commun.

[19] Ursini F, Maiorino M (2020). Lipid peroxidation and ferroptosis: The role of GSH and GPx4. Free Radic Biol Med.

[20] Doll S, Proneth B, Tyurina YY, Panzilius E, Kobayashi S, Ingold I (2017). ACSL4 dictates ferroptosis sensitivity by shaping cellular lipid composition. Nat Chem Biol.

[21] Lagrost L, Masson D (2022). The expanding role of lyso-phosphatidylcholine acyltransferase-3 (LPCAT3), a phospholipid remodeling enzyme, in health and disease. Curr Opin Lipidol.

[22] Yang WS, Kim KJ, Gaschler MM, Patel M, Shchepinov MS, Stockwell BR (2016). Peroxidation of polyunsaturated fatty acids by lipoxygenases drives ferroptosis. Proc Natl Acad Sci U S A.

[23] Couto N, Wood J, Barber J (2016). The role of glutathione reductase and related enzymes on cellular redox homoeostasis network. Free Radic Biol Med.

[24] Su Y, Zhao B, Zhou L, Zhang Z, Shen Y, Lv H (2020). Ferroptosis, a novel pharmacological mechanism of anti-cancer drugs. Cancer Lett.

[25] Kajarabille N, Latunde-Dada GO (2019). Programmed Cell-Death by Ferroptosis: Antioxidants as Mitigators. Int J Mol Sci.

[26] Xie Y, Zhu X, Liu P, Liu Y, Geng Y, Zhang L (2022). Xanthatin inhibits non-small cell lung cancer proliferation by breaking the redox balance. Drug Dev Res.

[27] Forcina GC, Dixon SJ (2019). GPX4 at the Crossroads of Lipid Homeostasis and Ferroptosis. Proteomics.

[28] Maiorino M, Conrad M, Ursini F (2018). GPx4, Lipid Peroxidation, and Cell Death: Discoveries, Rediscoveries, and Open Issues. Antioxid Redox Signal.

[29] Doll S, Freitas FP, Shah R, Aldrovandi M, da Silva MC, Ingold I (2019). FSP1 is a glutathione-independent ferroptosis suppressor. Nature.

[30] Bersuker K, Hendricks JM, Li Z, Magtanong L, Ford B, Tang PH (2019). The CoQ oxidoreductase FSP1 acts parallel to GPX4 to inhibit ferroptosis. Nature.

[31] Soula M, Weber RA, Zilka O, Alwaseem H, La K, Yen F (2020). Metabolic determinants of cancer cell sensitivity to canonical ferroptosis inducers. Nat Chem Biol.

[32] Kraft VAN, Bezjian CT, Pfeiffer S, Ringelstetter L, Muller C, Zandkarimi F (2020). GTP Cyclohydrolase 1/Tetrahydrobiopterin Counteract Ferroptosis through Lipid Remodeling. ACS Cent Sci.

[33] Wei X, Yi X, Zhu XH, Jiang DS (2020). Posttranslational Modifications in Ferroptosis. Oxid Med Cell Longev.

[34] Hu Q, Wei W, Wu D, Huang F, Li M, Li W (2022). Blockade of GCH1/BH4 Axis Activates Ferritinophagy to Mitigate the Resistance of Colorectal Cancer to Erastin-Induced Ferroptosis. Front Cell Dev Biol.

[35] Wang H, Liu C, Zhao Y, Gao G (2020). Mitochondria regulation in ferroptosis. Eur J Cell Biol.

[36] Mao C, Liu X, Zhang Y, Lei G, Yan Y, Lee H (2021). DHODH-mediated ferroptosis defence is a targetable vulnerability in cancer. Nature.

[37] Chen J, Li X, Ge C, Min J, Wang F (2022). The multifaceted role of ferroptosis in liver disease. Cell Death Differ.

[38] Gao M, Yi J, Zhu J, Minikes AM, Monian P, Thompson CB (2019). Role of Mitochondria in Ferroptosis. Mol Cell.

[39] Liu Y, Gu W (2022). p53 in ferroptosis regulation: the new weapon for the old guardian. Cell Death Differ.

[40] Anandhan A, Dodson M, Shakya A, Chen J, Liu P, Wei Y (2023). NRF2 controls iron homeostasis and ferroptosis through HERC2 and VAMP8. Sci Adv.

[41] Magesh S, Cai D (2022). Roles of YAP/TAZ in ferroptosis. Trends Cell Biol.

[42] Jiang H, Wang C, Zhang A, Li Y, Li J, Li Z (2022). ATF4 protects against sorafenib-induced cardiotoxicity by suppressing ferroptosis. Biomed Pharmacother.

[43] Vakifahmetoglu-Norberg H, Ouchida AT, Norberg E (2017). The role of mitochondria in metabolism and cell death. Biochem Biophys Res Commun.

[44] Gan B (2021). Mitochondrial regulation of ferroptosis. J Cell Biol.

[45] Bock FJ, Tait SWG (2020). Mitochondria as multifaceted regulators of cell death. Nat Rev Mol Cell Biol.

[46] Krainz T, Gaschler MM, Lim C, Sacher JR, Stockwell BR, Wipf P (2016). A Mitochondrial-Targeted Nitroxide Is a Potent Inhibitor of Ferroptosis. ACS Cent Sci.

[47] Su L, Zhang J, Gomez H, Kellum JA, Peng Z (2023). Mitochondria ROS and mitophagy in acute kidney injury. Autophagy.

[48] Su L, Zhang J, Gomez H, Kellum JA, Peng Z (2022). Mitochondria ROS and mitophagy in acute kidney injury. Autophagy.

[49] Otasevic V, Vucetic M, Grigorov I, Martinovic V, Stancic A (2021). Ferroptosis in Different Pathological Contexts Seen through the Eyes of Mitochondria. Oxid Med Cell Longev.

[50] Oakes SA, Papa FR (2015). The role of endoplasmic reticulum stress in human pathology. Annu Rev Pathol.

[51] Lu S, Wang XZ, He C, Wang L, Liang SP, Wang CC (2021). ATF3 contributes to brucine-triggered glioma cell ferroptosis via promotion of hydrogen peroxide and iron. Acta Pharmacol Sin.

[52] Li W, Li W, Leng Y, Xiong Y, Xia Z (2020). Ferroptosis Is Involved in Diabetes Myocardial Ischemia/Reperfusion Injury Through Endoplasmic Reticulum Stress. DNA Cell Biol.

[53] Chen PH, Wu J, Xu Y, Ding CC, Mestre AA, Lin CC (2021). Zinc transporter ZIP7 is a novel determinant of ferroptosis. Cell Death Dis.

[54] Chen MS, Wang SF, Hsu CY, Yin PH, Yeh TS, Lee HC (2017). CHAC1 degradation of glutathione enhances cystine-starvation-induced necroptosis and ferroptosis in human triple negative breast cancer cells via the GCN2-eIF2alpha-ATF4 pathway. Oncotarget.

[55] Lee YS, Lee DH, Choudry HA, Bartlett DL, Lee YJ (2018). Ferroptosis-Induced Endoplasmic Reticulum Stress: Cross-talk between Ferroptosis and Apoptosis. Mol Cancer Res.

[56] Hong SH, Lee DH, Lee YS, Jo MJ, Jeong YA, Kwon WT (2017). Molecular crosstalk between ferroptosis and apoptosis: emerging role of ER stress-induced p53-independent PUMA expression. Oncotarget.

[57] Kagan VE, Mao G, Qu F, Angeli JP, Doll S, Croix CS (2017). Oxidized arachidonic and adrenic PEs navigate cells to ferroptosis. Nat Chem Biol.

[58] Yang M, Li C, Sun L (2021). Mitochondria-Associated Membranes (MAMs): A Novel Therapeutic Target for Treating Metabolic Syndrome. Curr Med Chem.

[59] Li MD, Fu L, Lv BB, Xiang Y, Xiang HX, Xu DX (2022). Arsenic induces ferroptosis and acute lung injury through mtROS-mediated mitochondria-associated endoplasmic reticulum membrane dysfunction. Ecotoxicol Environ Saf.

[60] Ta N, Qu C, Wu H, Zhang D, Sun T, Li Y (2022). Mitochondrial outer membrane protein FUNDC2 promotes ferroptosis and contributes to doxorubicin-induced cardiomyopathy. Proc Natl Acad Sci U S A.

[61] Wang Y, Zhang M, Bi R, Su Y, Quan F, Lin Y (2022). ACSL4 deficiency confers protection against ferroptosis-mediated acute kidney injury. Redox Biol.

[62] Liang Y, Liu Z, Qu L, Wang Y, Zhou Y, Liang L (2022). Inhibition of the IRE1/JNK pathway in renal tubular epithelial cells attenuates ferroptosis in acute kidney injury. Front Pharmacol.

[63] Zhao C, Yu D, He Z, Bao L, Feng L, Chen L (2021). Endoplasmic reticulum stress-mediated autophagy activation is involved in cadmium-induced ferroptosis of renal tubular epithelial cells. Free Radic Biol Med.

[64] Chen X, Kang R, Kroemer G, Tang D (2021). Organelle-specific regulation of ferroptosis. Cell Death Differ.

[65] Alborzinia H, Ignashkova TI, Dejure FR, Gendarme M, Theobald J, Wolfl S (2018). Golgi stress mediates redox imbalance and ferroptosis in human cells. Commun Biol.

[66] Ballabio A, Bonifacino JS (2020). Lysosomes as dynamic regulators of cell and organismal homeostasis. Nat Rev Mol Cell Biol.

[67] Gao M, Monian P, Pan Q, Zhang W, Xiang J, Jiang X (2016). Ferroptosis is an autophagic cell death process. Cell Res.

[68] Mancias JD, Wang X, Gygi SP, Harper JW, Kimmelman AC (2014). Quantitative proteomics identifies NCOA4 as the cargo receptor mediating ferritinophagy. Nature.

[69] Torii S, Shintoku R, Kubota C, Yaegashi M, Torii R, Sasaki M (2016). An essential role for functional lysosomes in ferroptosis of cancer cells. Biochem J.

[70] Yang M, Chen P, Liu J, Zhu S, Kroemer G, Klionsky DJ (2019). Clockophagy is a novel selective autophagy process favoring ferroptosis. Sci Adv.

[71] Wang J, Liu Y, Wang Y, Sun L (2021). The Cross-Link between Ferroptosis and Kidney Diseases. Oxid Med Cell Longev.

[72] Rini BI, Campbell SC, Escudier B (2009). Renal cell carcinoma. Lancet.

[73] Jonasch E, Walker CL, Rathmell WK (2021). Clear cell renal cell carcinoma ontogeny and mechanisms of lethality. Nat Rev Nephrol.

[74] Chang K, Yuan C, Liu X (2021). Ferroptosis-Related Gene Signature Accurately Predicts Survival Outcomes in Patients With Clear-Cell Renal Cell Carcinoma. Front Oncol.

[75] Yang WS, SriRamaratnam R, Welsch ME, Shimada K, Skouta R, Viswanathan VS (2014). Regulation of ferroptotic cancer cell death by GPX4. Cell.

[76] Yang WH, Ding CC, Sun T, Rupprecht G, Lin CC, Hsu D (2019). The Hippo Pathway Effector TAZ Regulates Ferroptosis in Renal Cell Carcinoma. Cell Rep.

[77] Zou Y, Palte MJ, Deik AA, Li H, Eaton JK, Wang W (2019). A GPX4-dependent cancer cell state underlies the clear-cell morphology and confers sensitivity to ferroptosis. Nat Commun.

[78] Miess H, Dankworth B, Gouw AM, Rosenfeldt M, Schmitz W, Jiang M (2018). The glutathione redox system is essential to prevent ferroptosis caused by impaired lipid metabolism in clear cell renal cell carcinoma. Oncogene.

[79] Green YS, Ferreira Dos Santos MC, Fuja DG, Reichert EC, Campos AR, Cowman SJ (2022). ISCA2 inhibition decreases HIF and induces ferroptosis in clear cell renal carcinoma. Oncogene.

[80] Guo N (2022). Identification of ACSL4 as a biomarker and contributor of ferroptosis in clear cell renal cell carcinoma. Transl Cancer Res.

[81] Lu Y, Qin H, Jiang B, Lu W, Hao J, Cao W (2021). KLF2 inhibits cancer cell migration and invasion by regulating ferroptosis through GPX4 in clear cell renal cell carcinoma. Cancer Lett.

[82] Zhang W, Luo M, Xiong B, Liu X (2022). Upregulation of Metallothionein 1 G (MT1G) Negatively Regulates Ferroptosis in Clear Cell Renal Cell Carcinoma by Reducing Glutathione Consumption. J Oncol.

[83] Zheng Q, Li P, Zhou X, Qiang Y, Fan J, Lin Y (2021). Deficiency of the X-inactivation escaping gene KDM5C in clear cell renal cell carcinoma promotes tumorigenicity by reprogramming glycogen metabolism and inhibiting ferroptosis. Theranostics.

[84] Liu MZ, Kong N, Zhang GY, Xu Q, Xu Y, Ke P (2022). The critical role of ferritinophagy in human disease. Front Pharmacol.

[85] Mou Y, Wu J, Zhang Y, Abdihamid O, Duan C, Li B (2021). Low expression of ferritinophagy-related NCOA4 gene in relation to unfavorable outcome and defective immune cells infiltration in clear cell renal carcinoma. BMC Cancer.

[86] Kerins MJ, Milligan J, Wohlschlegel JA, Ooi A (2018). Fumarate hydratase inactivation in hereditary leiomyomatosis and renal cell cancer is synthetic lethal with ferroptosis induction. Cancer Sci.

[87] Markowitsch SD, Schupp P, Lauckner J, Vakhrusheva O, Slade KS, Mager R (2020). Artesunate Inhibits Growth of Sunitinib-Resistant Renal Cell Carcinoma Cells through Cell Cycle Arrest and Induction of Ferroptosis. Cancers (Basel).

[88] Yu R, Zhou Y, Shi S, Wang X, Huang S, Ren Y (2022). Icariside II induces ferroptosis in renal cell carcinoma cells by regulating the miR-324-3p/GPX4 axis. Phytomedicine.

[89] Shen H, Geng Z, Nie X, Liu T (2023). Erianin Induces Ferroptosis of Renal Cancer Stem Cells via Promoting ALOX12/P53 mRNA N6-methyladenosine Modification. J Cancer.

[90] Yangyun W, Guowei S, Shufen S, Jie Y, Rui Y, Yu R (2022). Everolimus accelerates Erastin and RSL3-induced ferroptosis in renal cell carcinoma. Gene.

[91] Du Y, Zhao HC, Zhu HC, Jin Y, Wang L (2021). Ferroptosis is involved in the anti-tumor effect of lycorine in renal cell carcinoma cells. Oncol Lett.

[92] Kellum JA, Romagnani P, Ashuntantang G, Ronco C, Zarbock A, Anders HJ (2021). Acute kidney injury. Nat Rev Dis Primers.

[93] Mercado MG, Smith DK, Guard EL (2019). Acute Kidney Injury: Diagnosis and Management. Am Fam Physician.

[94] Cabral BMI, Edding SN, Portocarrero JP, Lerma EV (2020). Rhabdomyolysis. Dis Mon.

[95] Vanholder R, Sukru Sever M, Lameire N (2021). Kidney problems in disaster situations. Nephrol Ther.

[96] Guerrero-Hue M, Garcia-Caballero C, Palomino-Antolin A, Rubio-Navarro A, Vazquez-Carballo C, Herencia C (2019). Curcumin reduces renal damage associated with rhabdomyolysis by decreasing ferroptosis-mediated cell death. FASEB J.

[97] Zarjou A, Bolisetty S, Joseph R, Traylor A, Apostolov EO, Arosio P (2013). Proximal tubule H-ferritin mediates iron trafficking in acute kidney injury. J Clin Invest.

[98] Sharfuddin AA, Molitoris BA (2011). Pathophysiology of ischemic acute kidney injury. Nat Rev Nephrol.

[99] Ni L, Yuan C, Wu X (2022). Targeting ferroptosis in acute kidney injury. Cell Death Dis.

[100] Ding C, Ding X, Zheng J, Wang B, Li Y, Xiang H (2020). miR-182-5p and miR-378a-3p regulate ferroptosis in I/R-induced renal injury. Cell Death Dis.

[101] Tonnus W, Meyer C, Steinebach C, Belavgeni A, von Massenhausen A, Gonzalez NZ (2021). Dysfunction of the key ferroptosis-surveilling systems hypersensitizes mice to tubular necrosis during acute kidney injury. Nat Commun.

[102] Zhao Z, Wu J, Xu H, Zhou C, Han B, Zhu H (2020). XJB-5-131 inhibited ferroptosis in tubular epithelial cells after ischemia-reperfusion injury. Cell Death Dis.

[103] Yan HF, Tuo QZ, Yin QZ, Lei P (2020). The pathological role of ferroptosis in ischemia/reperfusion-related injury. Zool Res.

[104] Qi Y, Hu M, Qiu Y, Zhang L, Yan Y, Feng Y (2023). Mitoglitazone ameliorates renal ischemia/reperfusion injury by inhibiting ferroptosis via targeting mitoNEET. Toxicol Appl Pharmacol.

[105] Du YW, Li XK, Wang TT, Zhou L, Li HR, Feng L (2023). Cyanidin-3-glucoside inhibits ferroptosis in renal tubular cells after ischemia/reperfusion injury via the AMPK pathway. Mol Med.

[106] Zhang J, Bi J, Ren Y, Du Z, Li T, Wang T (2021). Involvement of GPX4 in irisin's protection against ischemia reperfusion-induced acute kidney injury. J Cell Physiol.

[107] Wang Y, Quan F, Cao Q, Lin Y, Yue C, Bi R (2021). Quercetin alleviates acute kidney injury by inhibiting ferroptosis. J Adv Res.

[108] Jiang GP, Liao YJ, Huang LL, Zeng XJ, Liao XH (2021). Effects and molecular mechanism of pachymic acid on ferroptosis in renal ischemia reperfusion injury. Mol Med Rep.

[109] Xiao J, Yang Q, Zhang Y, Xu H, Ye Y, Li L (2021). Maresin conjugates in tissue regeneration-1 suppresses ferroptosis in septic acute kidney injury. Cell Biosci.

[110] Guo J, Chen L, Ma M (2023). Ginsenoside Rg1 Suppresses Ferroptosis of Renal Tubular Epithelial Cells in Sepsis-induced Acute Kidney Injury via the FSP1-CoQ10-NAD(P)H Pathway. Curr Med Chem.

[111] Guo L, Zhang T, Wang F, Chen X, Xu H, Zhou C (2021). Targeted inhibition of Rev-erb-alpha/beta limits ferroptosis to ameliorate folic acid-induced acute kidney injury. Br J Pharmacol.

[112] Martin-Sanchez D, Ruiz-Andres O, Poveda J, Carrasco S, Cannata-Ortiz P, Sanchez-Nino MD (2017). Ferroptosis, but Not Necroptosis, Is Important in Nephrotoxic Folic Acid-Induced AKI. J Am Soc Nephrol.

[113] Ozkok A, Edelstein CL (2014). Pathophysiology of cisplatin-induced acute kidney injury. Biomed Res Int.

[114] Zhou L, Yu P, Wang TT, Du YW, Chen Y, Li Z (2022). Polydatin Attenuates Cisplatin-Induced Acute Kidney Injury by Inhibiting Ferroptosis. Oxid Med Cell Longev.

[115] Deng L, Xiao M, Wu A, He D, Huang S, Deng T (2022). Se/Albumin Nanoparticles for Inhibition of Ferroptosis in Tubular Epithelial Cells during Acute Kidney Injury. ACS Applied Nano Materials.

[116] Hu Z, Zhang H, Yi B, Yang S, Liu J, Hu J (2020). VDR activation attenuate cisplatin induced AKI by inhibiting ferroptosis. Cell Death Dis.

[117] Xu Z, Zhang M, Wang W, Zhou S, Yu M, Qiu X (2023). Dihydromyricetin attenuates cisplatin-induced acute kidney injury by reducing oxidative stress, inflammation and ferroptosis. Toxicol Appl Pharmacol.

[118] Hu J, Gu W, Ma N, Fan X, Ci X (2022). Leonurine alleviates ferroptosis in cisplatin-induced acute kidney injury by activating the Nrf2 signalling pathway. Br J Pharmacol.

[119] Hung PH, Hsu YC, Chen TH, Lin CL (2021). Recent Advances in Diabetic Kidney Diseases: From Kidney Injury to Kidney Fibrosis. Int J Mol Sci.

[120] Feng Q, Liu D, Lu Y, Liu Z (2020). The Interplay of Renin-Angiotensin System and Toll-Like Receptor 4 in the Inflammation of Diabetic Nephropathy. J Immunol Res.

[121] Erekat NS (2022). Programmed Cell Death in Diabetic Nephropathy: A Review of Apoptosis, Autophagy, and Necroptosis. Med Sci Monit.

[122] Kim S, Kang SW, Joo J, Han SH, Shin H, Nam BY (2021). Characterization of ferroptosis in kidney tubular cell death under diabetic conditions. Cell Death Dis.

[123] Li S, Zheng L, Zhang J, Liu X, Wu Z (2021). Inhibition of ferroptosis by up-regulating Nrf2 delayed the progression of diabetic nephropathy. Free Radic Biol Med.

[124] Feng X, Wang S, Sun Z, Dong H, Yu H, Huang M (2021). Ferroptosis Enhanced Diabetic Renal Tubular Injury via HIF-1alpha/HO-1 Pathway in db/db Mice. Front Endocrinol (Lausanne).

[125] Wang Y, Bi R, Quan F, Cao Q, Lin Y, Yue C (2020). Ferroptosis involves in renal tubular cell death in diabetic nephropathy. Eur J Pharmacol.

[126] Wu K, Fei L, Wang X, Lei Y, Yu L, Xu W (2022). ZIP14 is involved in iron deposition and triggers ferroptosis in diabetic nephropathy. Metallomics.

[127] Zhang Q, Hu Y, Hu JE, Ding Y, Shen Y, Xu H (2021). Sp1-mediated upregulation of Prdx6 expression prevents podocyte injury in diabetic nephropathy via mitigation of oxidative stress and ferroptosis. Life Sci.

[128] Tan H, Chen J, Li Y, Li Y, Zhong Y, Li G (2022). Glabridin, a bioactive component of licorice, ameliorates diabetic nephropathy by regulating ferroptosis and the VEGF/Akt/ERK pathways. Mol Med.

[129] Jin T, Chen C (2022). Umbelliferone delays the progression of diabetic nephropathy by inhibiting ferroptosis through activation of the Nrf-2/HO-1 pathway. Food Chem Toxicol.

[130] Wang X, Li Q, Sui B, Xu M, Pu Z, Qiu T (2022). Schisandrin A from Schisandra chinensis Attenuates Ferroptosis and NLRP3 Inflammasome-Mediated Pyroptosis in Diabetic Nephropathy through Mitochondrial Damage by AdipoR1 Ubiquitination. Oxid Med Cell Longev.

[131] Chen J, Ou Z, Gao T, Yang Y, Shu A, Xu H (2022). Ginkgolide B alleviates oxidative stress and ferroptosis by inhibiting GPX4 ubiquitination to improve diabetic nephropathy. Biomed Pharmacother.

[132] Huang D, Shen P, Wang C, Gao J, Ye C, Wu F (2022). Calycosin plays a protective role in diabetic kidney disease through the regulation of ferroptosis. Pharm Biol.

[133] Feng Q, Yang Y, Qiao Y, Zheng Y, Yu X, Liu F (2023). Quercetin Ameliorates Diabetic Kidney Injury by Inhibiting Ferroptosis via Activating Nrf2/HO-1 Signaling Pathway. Am J Chin Med.

[134] Zhang S, Zhang S, Wang H, Chen Y (2023). Vitexin ameliorated diabetic nephropathy via suppressing GPX4-mediated ferroptosis. Eur J Pharmacol.

[135] Wang Y, Chang D, Zhao M, Chen M (2023). Dapagliflozin alleviates diabetic kidney disease via HIF1alpha/HO1 mediated ferroptosis. Antioxid Redox Signal.

[136] Huang J, Chen G, Wang J, Liu S, Su J (2022). Platycodin D regulates high glucose-induced ferroptosis of HK-2 cells through glutathione peroxidase 4 (GPX4). Bioengineered.

[137] Li Q, Meng X, Hua Q (2022). Circ ASAP2 decreased inflammation and ferroptosis in diabetic nephropathy through SOX2/SLC7A11 by miR-770-5p. Acta Diabetol.

[138] Jin J, Wang Y, Zheng D, Liang M, He Q (2022). A Novel Identified Circular RNA, mmu_mmu_circRNA_0000309, Involves in Germacrone-Mediated Improvement of Diabetic Nephropathy Through Regulating Ferroptosis by Targeting miR-188-3p/GPX4 Signaling Axis. Antioxid Redox Signal.

[139] Wang H, Liu D, Zheng B, Yang Y, Qiao Y, Li S (2023). Emerging Role of Ferroptosis in Diabetic Kidney Disease: Molecular Mechanisms and Therapeutic Opportunities. Int J Biol Sci.

[140] Li LX, Fan LX, Zhou JX, Grantham JJ, Calvet JP, Sage J (2017). Lysine methyltransferase SMYD2 promotes cyst growth in autosomal dominant polycystic kidney disease. J Clin Invest.

[141] Agborbesong E, Li LX, Li L, Li X (2022). Molecular Mechanisms of Epigenetic Regulation, Inflammation, and Cell Death in ADPKD. Front Mol Biosci.

[142] Schreiber R, Buchholz B, Kraus A, Schley G, Scholz J, Ousingsawat J (2019). Lipid Peroxidation Drives Renal Cyst Growth In Vitro through Activation of TMEM16A. J Am Soc Nephrol.

[143] Zhang X, Li LX, Ding H, Torres VE, Yu C, Li X (2021). Ferroptosis Promotes Cyst Growth in Autosomal Dominant Polycystic Kidney Disease Mouse Models. J Am Soc Nephrol.

[144] Barinotti A, Radin M, Cecchi I, Foddai SG, Rubini E, Roccatello D (2022). Serum Biomarkers of Renal Fibrosis: A Systematic Review. Int J Mol Sci.

[145] Panizo S, Martinez-Arias L, Alonso-Montes C, Cannata P, Martin-Carro B, Fernandez-Martin JL (2021). Fibrosis in Chronic Kidney Disease: Pathogenesis and Consequences. Int J Mol Sci.

[146] Romagnani P, Remuzzi G, Glassock R, Levin A, Jager KJ, Tonelli M (2017). Chronic kidney disease. Nat Rev Dis Primers.

[147] Liu Y, Wang J (2022). Ferroptosis, a Rising Force against Renal Fibrosis. Oxid Med Cell Longev.

[148] Zhang B, Chen X, Ru F, Gan Y, Li B, Xia W (2021). Liproxstatin-1 attenuates unilateral ureteral obstruction-induced renal fibrosis by inhibiting renal tubular epithelial cells ferroptosis. Cell Death Dis.

[149] Yang L, Guo J, Yu N, Liu Y, Song H, Niu J (2020). Tocilizumab mimotope alleviates kidney injury and fibrosis by inhibiting IL-6 signaling and ferroptosis in UUO model. Life Sci.

[150] Li J, Yang J, Zhu B, Fan J, Hu Q, Wang L (2022). Tectorigenin protects against unilateral ureteral obstruction by inhibiting Smad3-mediated ferroptosis and fibrosis. Phytother Res.

[151] Wang J, Wang Y, Liu Y, Cai X, Huang X, Fu W (2022). Ferroptosis, a new target for treatment of renal injury and fibrosis in a 5/6 nephrectomy-induced CKD rat model. Cell Death Discov.

[152] Gong S, Zhang A, Yao M, Xin W, Guan X, Qin S (2023). REST contributes to AKI-to-CKD transition through inducing ferroptosis in renal tubular epithelial cells. JCI Insight.

[153] Xiong D, Hu W, Han X, Cai Y (2023). Rhein Inhibited Ferroptosis and EMT to Attenuate Diabetic Nephropathy by Regulating the Rac1/NOX1/beta-Catenin Axis. Front Biosci (Landmark Ed).

[154] Zhu B, Ni Y, Gong Y, Kang X, Guo H, Liu X (2023). Formononetin ameliorates ferroptosis-associated fibrosis in renal tubular epithelial cells and in mice with chronic kidney disease by suppressing the Smad3/ATF3/SLC7A11 signaling. Life Sci.

[155] Li X, Zou Y, Xing J, Fu YY, Wang KY, Wan PZ (2020). Pretreatment with Roxadustat (FG-4592) Attenuates Folic Acid-Induced Kidney Injury through Antiferroptosis via Akt/GSK-3beta/Nrf2 Pathway. Oxid Med Cell Longev.

[156] Mejia-Vilet JM, Malvar A, Arazi A, Rovin BH (2022). The lupus nephritis management renaissance. Kidney Int.

[157] Wang W, Lin Z, Feng J, Liang Q, Zhao J, Zhang G (2022). Identification of ferroptosis-related molecular markers in glomeruli and tubulointerstitium of lupus nephritis. Lupus.

[158] Alli Abdel A (2022). DD, Elshika Ahmed, Morel Laurence, Conrad Marcus, Proneth Bettina, Clapp William, Atkinson Carl, Segal Mark, Searcy Louis A, Denslow Nancy D, Bolisetty Subhashini, Mehrad Borna, Scindia Yogesh. Kidney tubular epithelial cell ferroptosis links glomerular injury to tubulointerstitial pathology in lupus nephritis. bioRxiv - Immunology.

[159] Du Y, Cheng T, Liu C, Zhu T, Guo C, Li S (2023). IgA Nephropathy: Current Understanding and Perspectives on Pathogenesis and Targeted Treatment. Diagnostics (Basel).

[160] Tian ZY, Li Z, Chu L, Liu Y, He JR, Xin Y (2023). Iron metabolism and chronic inflammation in IgA nephropathy. Ren Fail.

[161] Wu J, Shao X, Shen J, Lin Q, Zhu X, Li S (2022). Downregulation of PPARalpha mediates FABP1 expression, contributing to IgA nephropathy by stimulating ferroptosis in human mesangial cells. Int J Biol Sci.

[162] Qiu Y, Cao Y, Cao W, Jia Y, Lu N (2020). The Application of Ferroptosis in Diseases. Pharmacol Res.

[163] Bayir H, Dixon SJ, Tyurina YY, Kellum JA, Kagan VE (2023). Ferroptotic mechanisms and therapeutic targeting of iron metabolism and lipid peroxidation in the kidney. Nat Rev Nephrol.

[164] Song SH, Han D, Park K, Um JE, Kim S, Ku M (2023). Bone morphogenetic protein-7 attenuates pancreatic damage under diabetic conditions and prevents progression to diabetic nephropathy via inhibition of ferroptosis. Front Endocrinol (Lausanne).

[165] Yang L, Liu Y, Zhou S, Feng Q, Lu Y, Liu D Novel insight into ferroptosis in kidney diseases. Am J Nephrol. 2023: 1.

